# Community-Level Differences in the Microbiome of Healthy Wild Mallards and Those Infected by Influenza A Viruses

**DOI:** 10.1128/mSystems.00188-16

**Published:** 2017-02-28

**Authors:** Holly H. Ganz, Ladan Doroud, Alana J. Firl, Sarah M. Hird, Jonathan A. Eisen, Walter M. Boyce

**Affiliations:** a Genome Center, University of California, Davis, Davis, California, USA; b Department of Computer Science, University of California, Davis, Davis, California, USA; c Department of Molecular and Cell Biology, University of Connecticut, Storrs, Connecticut, USA; d Department of Pathology, Microbiology, and Immunology, School of Veterinary Medicine, University of California, Davis, Davis, California, USA; G.W. Hooper Research Foundation

**Keywords:** influenza, mallard, microbiome, network modeling, machine learning, biomarkers

## Abstract

Waterfowl, especially ducks and geese, are primary reservoirs for influenza A viruses (IAVs) that evolve and emerge as important pathogens in domestic animals and humans. In contrast to humans, where IAVs infect the respiratory tract and cause significant morbidity and mortality, IAVs infect the gastrointestinal tract of waterfowl and cause little or no pathology and are spread by fecal-oral transmission. For this reason, we examined whether IAV infection is associated with differences in the cloacal microbiome of mallards (*Anas platyrhyncos*), an important host of IAVs in North America and Eurasia. We characterized bacterial community composition by sequencing the V4 region of 16S rRNA genes. IAV-positive mallards had lower species diversity, richness, and evenness than IAV-negative mallards. Operational taxonomic unit (OTU) cooccurrence patterns were also distinct depending on infection status. Network analysis showed that IAV-positive mallards had fewer significant cooccurring OTUs and exhibited fewer coassociation patterns among those OTUs than IAV-negative mallards. These results suggest that healthy mallards have a more robust and complex cloacal microbiome. By combining analytical approaches, we identified 41 bacterial OTUs, primarily representatives of *Streptococcus* spp., *Veillonella dispar*, and *Rothia mucilaginosa*, contributing to the observed differences. This study found that IAV-infected wild mallards exhibited strong differences in microbiome composition relative to noninfected mallards and identified a concise set of putative biomarker OTUs. Using Random Forest, a supervised machine learning method, we verified that these 41 bacterial OTUs are highly predictive of infection status.

**IMPORTANCE** Seasonal influenza causes 3 to 5 million severe illnesses and 250,000 to 500,000 human deaths each year. While pandemic influenza viruses emerge only periodically, they can be devastating—for example, the 1918 H1N1 pandemic virus killed more than 20 million people. IAVs infect the respiratory tract and cause significant morbidity and mortality in humans. In contrast, IAVs infect the gastrointestinal tract of waterfowl, producing little pathology. Recent studies indicated that viruses can alter the microbiome at the respiratory and gastrointestinal mucosa, but there are no reports of how the microbiota of the natural host of influenza is affected by infection. Here we find that the mallard microbiome is altered during IAV infection. Our results suggest that detailed examination of humans and animals infected with IAVs may reveal individualized microbiome profiles that correspond to health and disease. Moreover, future studies should explore whether the altered microbiome facilitates maintenance and transmission of IAVs in waterfowl populations.

## Abstract (precis)

Seasonal influenza causes 3 to 5 million severe illnesses and 250,000 to 500,000 human deaths each year. While pandemic influenza viruses emerge only periodically, they can be devastating—for example, the 1918 H1N1 pandemic virus killed more than 20 million people. IAVs infect the respiratory tract and cause significant morbidity and mortality in humans. In contrast, IAVs infect the gastrointestinal tract of waterfowl, producing little pathology. Recent studies indicated that viruses can alter the microbiome at the respiratory and gastrointestinal mucosa, but there are no reports of how the microbiota of the natural host of influenza is affected by infection. Here we find that the mallard microbiome is altered during IAV infection. Our results suggest that detailed examination of humans and animals infected with IAVs may reveal individualized microbiome profiles that correspond to health and disease. Moreover, future studies should explore whether the altered microbiome facilitates maintenance and transmission of IAVs in waterfowl populations.

## INTRODUCTION

Mallards (*Anas platyrhyncos*) are an important natural reservoir in North America and Eurasia for many subtypes of influenza A viruses (IAVs), the group of influenza viruses mainly responsible for seasonal and pandemic influenza in humans (1). Waterfowl (ducks, geese, and swans in the avian family *Anatidae*) are the primary reservoir of naturally occurring IAVs (2, 6). In humans and domestic animals (poultry, swine, horses, dogs, etc.), IAVs contain gene segments that trace back to a wild avian origin (3, 4). Recent concern over the emergence of new pandemic IAVs led to intensive IAV surveillance in waterfowl, especially of dabbling ducks in the genus *Anas*, which revealed that IAV prevalence may reach 40%, especially in young (hatch year) birds (58–61, 65). In contrast to humans, where IAVs infect the respiratory tract and cause significant morbidity and mortality, IAVs infect the gastrointestinal tract of waterfowl and cause little or no pathology (5) and are spread by fecal-oral transmission (6).

Here we investigated the bacterial microbiome in wild, juvenile mallards and tested for associations with IAV infection. The microbiome was first defined by Whipps et al. (7) as a characteristic microbial community (including bacteria, archaea, fungi, the various single-celled eukaryotes generally referred to as protists, and viruses) occupying a distinct habitat. For a variety of reasons, most microbiome research to date has focused on the bacterial portion of these communities. The application of contemporary sequencing methods to characterize diversity in the microbiome has revolutionized the study of interactions between microbial inhabitants of the microbiome in general as well as the study of interactions between members of the microbiome and particular pathogens (see, e.g., references 8–12). Identification of potential effects of IAV infection on the microbiome of mallards (a natural reservoir) should contribute to a greater understanding of how these viruses are maintained and transmitted in nature.

When a mallard ingests IAV from contaminated material in the environment, the process of infection requires a virus to pass from the gut lumen through mucus, which presents a potent physical and immunological barrier to microbial invasion (13, 14, 15). In birds and mammals, mucus is composed primarily of mucins produced by underlying epithelial cells and is divided into two layers: an outer layer that typically contains a diverse community of resident microbes and an inner “protected” layer that has a low density of microbes except during pathogen invasion (15). In some cases, the microbiome of the mucus layer may affect pathogen attachment to host cells; for example, glycan structures on the surface of the bacterium *Enterobacter cloacae* in the human gut microbiome may facilitate the attachment of human norovirus to host cells (16). In addition, some orally transmitted viruses, including poliovirus and reovirus, may manipulate the bacterial microbiome to promote their transmission across the mucosal interface (reviewed in reference 10). Along with its associated microbiota and any potential pathogens trapped in the mucus layer, mucus is continuously expelled from the body. Once IAVs have crossed the mucosal barrier in the intestinal tract of waterfowl, they amplify inside host epithelial cells and are then shed into the environment and transmitted to new hosts. Free-ranging mallards typically shed IAVs in feces for about 6 days after infection (5, 17).

In this study, we analyzed the relationship between infection by low-pathogenicity IAVs and the composition and species cooccurrence patterns of bacteria inhabiting the cloacal microbiome of juvenile mallards. We hypothesized that IAV infection would be associated with an altered microbiome. To investigate this, we identified members of the microbiome that contribute to the observed associations. We compared bacterial community composition characteristics using standard approaches (alpha and beta diversity; see Materials and Methods), and, to assess the relationships between groups of taxa, we analyzed bacterial operational taxonomic unit (OTU) cooccurrence patterns. Given that groups of bacterial taxa are often both functionally and phylogenetically related, it is useful to assess how infection might affect interdependencies between bacterial OTUs. In order to investigate potential interactions and relationships among different microbial taxa, we analyzed their cooccurrence relationships using a network. Network analysis of significant cooccurrence structures of microbial taxa has helped decipher complex microbial dynamics such as groups of taxa that are metabolically linked or are genetically related (18).

Although several studies have focused on IAVs and the microbiome in humans and mice (19–25), we believe that this is the first study to have investigated the relationship between IAV infection and the microbiome of a wild bird that is an important primary reservoir.

## RESULTS

Cloacal swabs were collected from 122 juvenile mallards in the Suisun Bay area in California in the United States between 2009 and 2013. All mallards sampled in this study were apparently healthy at the time of sampling. We performed influenza testing and microbiome analysis on the cloacal swabs.

**FIG 1:**
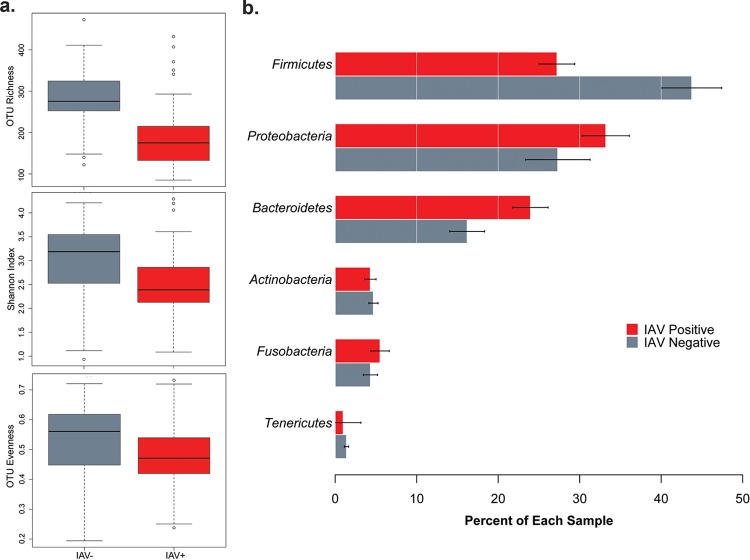
(a) IAV-positive (IAV^+^) and IAV^−^ mallards also differed in OTU richness, Shannon index, and OTU evenness results. (b) Proportions of bacterial phyla in the cloacal microbiome differed between IAV^+^ and IAV^−^ mallards.

OTU richness levels in cloacal samples differed based on infection status (*t* = 7.20, *df* = 94.8, *P* < 0.00001) (Fig. 1a). After rarefaction to 5,000 reads per sample and removal of OTUs that were unidentified at the kingdom level, IAV-negative (here “IAV^−^”) mallards had 1.5 times as many OTUs per individual (282 OTUs) as IAV-positive (IAV^+^) mallards (187 OTUs; Fig. 1a). OTU diversity (based on Shannon index values) was greater in IAV^−^ mallards than in IAV^+^ mallards (*t* = 3.77, *df* = 78.83, *P* = 0.0003) (Fig. 1a), and OTU evenness was greater in IAV^−^ mallards than in IAV^+^ mallards (*t* = 2.53, *df* = 76.45, *P* = 0.014) (Fig. 1a).

Representatives of the bacterial phyla *Firmicutes*, *Proteobacteria*, *Bacteroidetes*, *Actinobacteria*, *Fusobacteria*, and *Tenericutes* were detected in all of the samples, comprising averages of 44%, 27%, 16%, 5%, 4%, and 1.4% in IAV^−^ mallards and 27%, 33%, 24%, 4%, 5.5%, and 1% in IAV^+^ mallards, respectively (Fig. 1b). Infection status was correlated with large differences in the proportions of bacterial OTUs, particularly within the phyla *Firmicutes*, *Proteobacteria*, and *Bacteroidetes* (Fig. 1b).

The overall composition of cloacal bacteria in IAV^+^ mallards differed significantly from that in IAV^−^ mallards (permutational multivariate analysis of variance [PERMANOVA]: *F*^1,114^ = 11.2; *P* < 0.001), even though the community composition was influenced by the year of sampling (PERMANOVA: *F*^1,114^ = 3: 28; *P* < 0.001), and the interaction between influenza infection status and year of sampling was also significant (PERMANOVA: *F*^1,114^ = 3.05; *P* < 0.002). The microbiome of mallard males did not differ from that of females (PERMANOVA: *F*^2,114^ = 1.14; *P* = 0.24). Despite the effect of the year data, we detected significant clustering based on infection status (see Fig. S1 in the supplemental material).

10.1128/mSystems.00188-16.1 FIG S1  PCoA plots of IAV^+^ and IAV^−^ mallards. The top row shows PCoA results calculated on log-scaled abundance values; the bottom row shows PCoA results calculated on binarized presence/absence versions of the same data. (a) A total of 10,363 OTUs found in the data after rarefaction (5,000 reads per sample). (b) A total of 10,363 OTUs minus 85 OTUs that were identified as significant by the G-test. (c) A total of 10,363 OTUs minus 47 OTUs that were identified based on the DIROM. Download FIG S1, PDF file, 0.4 MB. Copyright © 2017 Ganz et al. 2017 Ganz et al. This content is distributed under the terms of the Creative Commons Attribution 4.0 International license.

### Infected mallards only.

Among IAV^+^ mallards, there was a significant effect of IAV subtype (based on the levels of the surface proteins hemagglutinin [HA] and neuraminidase [NA]) on bacterial community composition (*F*^1,69^ = 1.35, *P* < 0.004). In addition, we detected a significant effect of year of sampling (*F*^1,69^ = 3.03, *P* < 0.004), likely because some subtypes occurred only in certain years, and the interaction between HA and NA subtype and the year that the mallards were sampled was significant (*F*^1,69^ = 2.14, *P* = 0.007).

Using the DIROM (Difference in the Relative Occurrence Metric; see Materials and Methods) method, we identified all OTUs that had a greater than 50% difference in how frequently they occurred in the two groups. This resulted in 47 OTUs (see Table S1 in the supplemental material), all of which occurred in greater frequency in the IAV^−^ mallards. These OTUs belonged to the phyla *Tenericutes* (*n* = 1), *Proteobacteria* (*n* = 4), *Firmicutes* (*n* = 31), *Bacteroidetes* (*n* = 5), and *Actinobacteria* (*n* = 6). According to the results obtained with a Quantitative Insights in Microbial Ecology (QIIME) taxonomy assignment script (assign_taxonomy.py), 21 of the 47 OTUs belonged to the genus *Streptococcus*, 5 were representatives of the species *Rothia mucilaginosa*, and 5 were representatives of the species *Veillonella dispar*. The largest difference belonged to the single OTU from the *Tenericutes*, representing a mycoplasma, whose examples differed by 92.1% between the groups and, like the other OTUs, occurred more frequently in IAV^−^ mallards.

10.1128/mSystems.00188-16.2 TABLE S1  OTUs that differed by more than 50% in relative occurrence levels between IAV^+^ and IAV^−^ mallards (difference in relative occurrence metric [DIROM]). Download TABLE S1, DOCX file, 0.1 MB. Copyright © 2017 Ganz et al. 2017 Ganz et al. This content is distributed under the terms of the Creative Commons Attribution 4.0 International license.

We also used the G-test (see Materials and Methods) to identify 85 OTUs that differed in abundance between IAV^−^ and IAV^+^ mallards (Table S2). IAV^−^ mallards had higher abundances in 51 OTUs in *Firmicutes*, particularly in representatives of *Streptococcus* (*n* = 31 OTUs) and *Veillonella* (*n* = 14 OTUs), as well as OTUs in other phyla, including *Actinobacteria* (*n* = 10), *Bacteroidetes* (*n* = 10), *Proteobacteria* (*n* = 5), *Tenericutes* (*n* = 4), and *Fusobacteria* (*n* = 3). Only one OTU (in phylum *Actinobacteria*, family *Micrococcaceae*) was significantly enriched in the IAV^+^ mallards compared to IAV^−^ individuals.

10.1128/mSystems.00188-16.3 TABLE S2  A total of 85 OTUs differed significantly in mean abundance levels between IAV^+^ and IAV^−^ mallards based on the G-test (*P* value corrected for multiple comparisons with Bonferroni method). Download TABLE S2, DOCX file, 0.1 MB. Copyright © 2017 Ganz et al. 2017 Ganz et al. This content is distributed under the terms of the Creative Commons Attribution 4.0 International license.

### Network analysis.

The resulting bacterial networks (see Materials and Methods) both consisted of 674 nodes (OTUs) with 112,488 non-zero-weighted edges in IAV^+^ and 132,875 edges in IAV^−^ networks with average node connections (i.e., the average number of edges incident to each node) of 333.79 and 394.02, respectively. The average values for weighted degree (i.e., sum of all the weights for incident edges) were 13.12 for IAV^+^ and 29.89 for IAV^−^. These results show that OTUs in the IAV^+^ group had a lower number of connections in total and that the edges had relatively much lower weights. Thus, the OTUs in IAV^+^ mallards cooccurred in fewer IAV^+^ mallards than IAV^−^ mallards.

**FIG 2:**
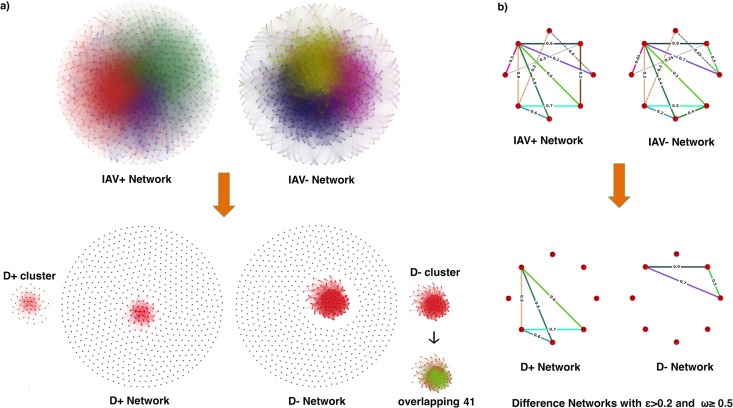
(a) Networks of cooccurrence patterns for OTUs found in mallards that tested either positive for IAV (IAV^+^) or negative for IAV (IAV^−^). Networks of D^+^ and D^−^ data were constructed from the IAV^+^ and IAV^−^ networks by adding edges that differed in edge weight by more than a threshold ε value of 0.2 from those of their D networks. Once D networks were created, we applied an edge threshold value of 0.5 to remove the edges with low edge weights. The clusters obtained for the D^+^ and D^−^ networks with the overlapping 41 are shown next to the difference networks and are colored (in D^−^ cluster). (b) Toy graph illustrating our method.

**FIG 3:**
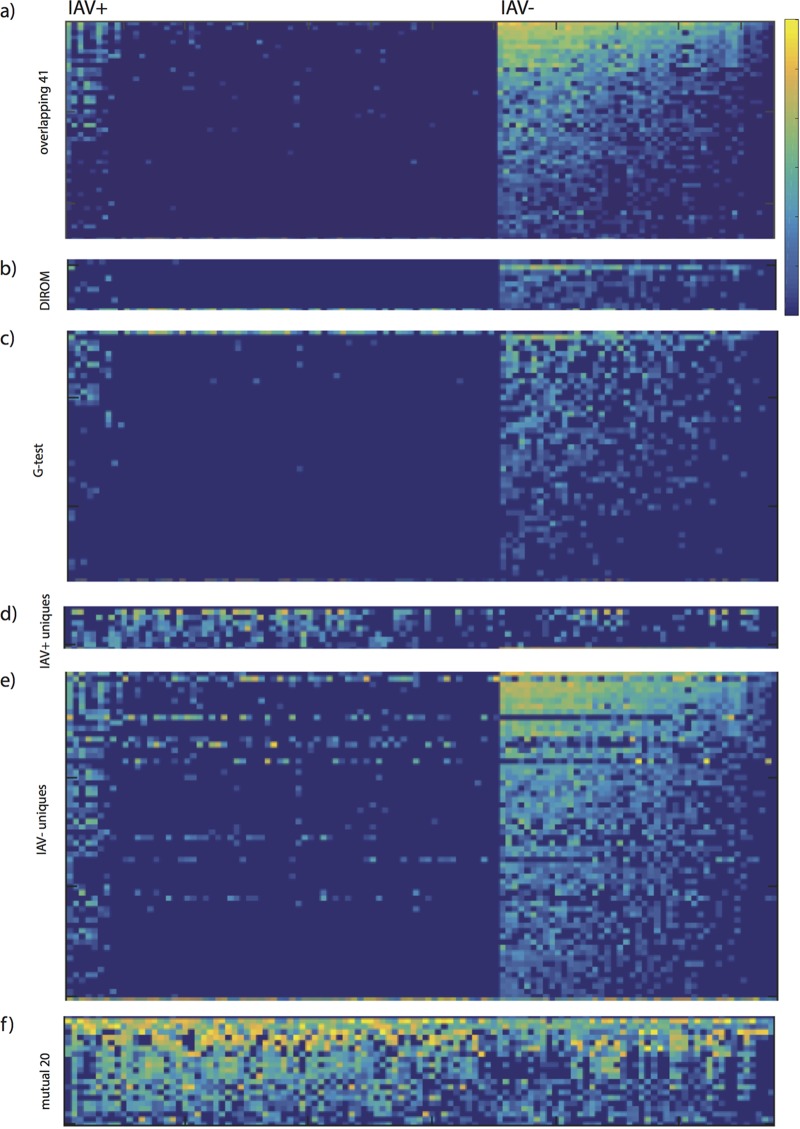
Log-scaled abundances of OTUs under different IAV conditions. (a) A total of 41 overlapping OTUs. (b) A total of 8 DIROM OTUs, not including the overlapping 41. (c) A total of 46 G-test OTUs, not including the overlapping 41. (d) A total of 7 OTUs that were uniquely found to be highly cooccurring in IAV^*+*^ networks. (e) A total of 80 OTUs that were uniquely found to be highly cooccurring in IAV^*−*^ networks. (f) A total of 20 OTUs that were mutually significant under both IAV conditions.

To ensure that we were detecting OTUs associated with infection status (rather than clusters idiosyncratic to individuals), we filtered the OTU table to include only those OTUs that appeared in two or more samples per IAV condition, resulting in 674 OTUs. We then characterized OTU cooccurrence networks across the 674 OTUs in the IAV^+^ and IAV^−^ mallards and compared the IAV^+^ and IAV^−^ networks to generate difference networks (D^+^ and D^−^; see Materials and Methods). Briefly, the cooccurrences that were at higher levels in IAV^+^ were assigned to D^+^, and the cooccurrences that were at higher levels in IAV^−^ were assigned to D^−^. The assumption is that the clusters of OTUs highly cooccurring in one group (D^+^ or D^−^) but not the other were infection dependent. The resulting difference networks both consisted of 674 nodes (OTUs) with 344 edges in D^+^ and 6,702 edges in D^−^ networks with average node connections of 1.02 and 19.8, respectively. We found that the network of D^+^ mallards had fewer edges and thus lower network density than the network of D^−^ mallards. The density values were 0.002 in D^+^ and 0.03 in D^−^, showing proportionally larger numbers of existing connections in D. In order to identify core communities of bacteria that were associated with IAV infection, we set a threshold for edge weights (see Materials and Methods) to exclude weak connections and then decomposed our D^+^ and D^−^ networks into modules (i.e., groups of nodes that were more densely connected to one another than to the rest of the network) (26) to find significant community differences in cooccurrence patterns between IAV^+^ and IAV^−^ mallards. Both D^+^ and D^−^ resulted in the formation of one cluster by the use of the Chinese whispers clustering method (27) and Markov cluster algorithm (MCL) clustering (28) with 27 and 80 nodes, respectively. A visualization of these clusters is shown in Fig. 2. Twenty of these OTUs were mutual (shared) between the two groups (mutual 20). The mutual 20 exhibited similar abundance patterns across all the samples regardless of the infection group (Fig. 3) and may be part of the core microbiome of the mallard. In addition to the mutual 20 OTUs, we found 7 OTUs that were uniquely identified by the D^+^ cluster and we found 60 OTUs that were uniquely identified by the D^−^ cluster. The set of OTUs that were uniquely identified in D^+^ may be indicative of a community of bacteria that are activated in the presence of infection. This approach allows us to identify those OTUs that were uniquely identified in the D^−^ cluster (and therefore absent in the D^+^ cluster) and may reflect bacteria that are diminished in population during infection. The obtained cluster from D^−^ was bigger and more densely connected, whereas the D^+^ cluster had fewer OTUs and weaker connections. Therefore, the cluster from D^−^ may represent stronger patterns of coassociation between OTUs.

In the final step of network modeling, we used the difference networks to identify clusters that were highly correlated with groups of mallards based on their infection status (Table S3). Notably, 41 of the OTUs identified by network analysis overlapped the OTUs identified by G-test and DIROM (Fig. 3; Table 1). All of these OTUs were contained in the clusters retrieved from the D^−^ network. We studied the distribution of those 41 OTUs across the clusters and observed that all of them were contained in one cluster and had high edge weights (Fig. 2).

10.1128/mSystems.00188-16.4 TABLE S3  Taxonomic assignments for OTUs identified in network modeling as significantly clustered. Download TABLE S3, DOCX file, 0.1 MB. Copyright © 2017 Ganz et al. 2017 Ganz et al. This content is distributed under the terms of the Creative Commons Attribution 4.0 International license.

**TABLE 1:** Taxonomic information for the 41 OTUs identified as contributing to observed differences in IAV^−^ and IAV^+^ juvenile mallards based on the intersection of the DIROM, G-test, and network analysis results^a^

OTU ID	Phylum	Class	Order	Family	Genus	Species
4466006	*Actinobacteria*	*Actinobacteria*	*Actinomycetales*	*Micrococcaceae*	*Rothia*	*dentocariosa*
866280	*Actinobacteria*	*Actinobacteria*	*Actinomycetales*	*Micrococcaceae*	*Rothia*	*mucilaginosa*
1017181	*Actinobacteria*	*Actinobacteria*	*Actinomycetales*	*Micrococcaceae*	*Rothia*	*mucilaginosa*
4294457	*Actinobacteria*	*Actinobacteria*	*Actinomycetales*	*Micrococcaceae*	*Rothia*	*mucilaginosa*
4411138	*Actinobacteria*	*Actinobacteria*	*Actinomycetales*	*Micrococcaceae*	*Rothia*	*mucilaginosa*
269907	*Bacteroidetes*	*Bacteroidia*	*Bacteroidales*	(*Prevotellaceae*)	(*Prevotella*)	
4423790	*Bacteroidetes*	*Bacteroidia*	*Bacteroidales*	*Porphyromonadaceae*	*Porphyromonas*	*endodontalis*
4321559	*Bacteroidetes*	*Bacteroidia*	*Bacteroidales*	*Porphyromonadaceae*	*Porphyromonas*	
4307391	*Bacteroidetes*	*Bacteroidia*	*Bacteroidales*	*Prevotellaceae*	*Prevotella*	*melaninogenica*
4446902	*Firmicutes*	*Bacilli*	*Gemellales*	*Gemellaceae*		
92535	*Firmicutes*	*Bacilli*	*Lactobacillales*	*Streptococcaceae*	*Streptococcus*	
298862	*Firmicutes*	*Bacilli*	*Lactobacillales*	*Streptococcaceae*	*Streptococcus*	
513646	*Firmicutes*	*Bacilli*	*Lactobacillales*	*Streptococcaceae*	*Streptococcus*	
526804	*Firmicutes*	*Bacilli*	*Lactobacillales*	*Streptococcaceae*	*Streptococcus*	
536866	*Firmicutes*	*Bacilli*	*Lactobacillales*	*Streptococcaceae*	*Streptococcus*	
584109	*Firmicutes*	*Bacilli*	*Lactobacillales*	*Streptococcaceae*	*Streptococcus*	
864465	*Firmicutes*	*Bacilli*	*Lactobacillales*	*Streptococcaceae*	*Streptococcus*	
1000547	*Firmicutes*	*Bacilli*	*Lactobacillales*	*Streptococcaceae*	*Streptococcus*	
2953981	*Firmicutes*	*Bacilli*	*Lactobacillales*	*Streptococcaceae*	*Streptococcus*	
3384047	*Firmicutes*	*Bacilli*	*Lactobacillales*	*Streptococcaceae*	*Streptococcus*	
4306048	*Firmicutes*	*Bacilli*	*Lactobacillales*	*Streptococcaceae*	*Streptococcus*	
4307484	*Firmicutes*	*Bacilli*	*Lactobacillales*	*Streptococcaceae*	*Streptococcus*	
4309301	*Firmicutes*	*Bacilli*	*Lactobacillales*	*Streptococcaceae*	*Streptococcus*	
4424239	*Firmicutes*	*Bacilli*	*Lactobacillales*	*Streptococcaceae*	*Streptococcus*	
4425214	*Firmicutes*	*Bacilli*	*Lactobacillales*	*Streptococcaceae*	*Streptococcus*	
4439603	*Firmicutes*	*Bacilli*	*Lactobacillales*	*Streptococcaceae*	*Streptococcus*	
4442130	*Firmicutes*	*Bacilli*	*Lactobacillales*	*Streptococcaceae*	*Streptococcus*	
4455767	*Firmicutes*	*Bacilli*	*Lactobacillales*	*Streptococcaceae*	*Streptococcus*	
NR.OTU326	*Firmicutes*	*Bacilli*	*Lactobacillales*	*Streptococcaceae*	*Streptococcus*	
271159	*Firmicutes*	*Bacilli*	*Lactobacillales*			
1696853	*Firmicutes*	*Bacilli*	*Lactobacillales*			
1061772	*Firmicutes*	*Bacilli*				
4316391	*Firmicutes*	*Clostridia*	*Clostridiales*	*Veillonellaceae*	*Veillonella*	*dispar*
4318671	*Firmicutes*	*Clostridia*	*Clostridiales*	*Veillonellaceae*	*Veillonella*	*dispar*
4410401	*Firmicutes*	*Clostridia*	*Clostridiales*	*Veillonellaceae*	*Veillonella*	*dispar*
4453501	*Firmicutes*	*Clostridia*	*Clostridiales*	*Veillonellaceae*	*Veillonella*	*dispar*
4458959	*Firmicutes*	*Clostridia*	*Clostridiales*	*Veillonellaceae*	*Veillonella*	*parvula*
4318122	*Proteobacteria*	*Gammaproteobacteria*	*Pasteurellales*	*Pasteurellaceae*	*Actinobacillus*	*porcinus*
70728	*Proteobacteria*	*Gammaproteobacteria*	*Pasteurellales*	*Pasteurellaceae*	*Aggregatibacter*	*pneumotropica*
4477696	*Proteobacteria*	*Gammaproteobacteria*	*Pasteurellales*	*Pasteurellaceae*	*Haemophilus*	
NR.OTU97	*Tenericutes*	*Mollicutes*	*Mycoplasmatales*	*Mycoplasmataceae*	*Mycoplasma*	

a ID, identifier; NR, new reference.

To confirm whether these OTUs are predictive of infection status, we also used the Random Forest model, which is a supervised machine-learning technique (29). The 3-fold cross-validation approach was applied to estimate the accuracy of the classifier. We assigned OTUs as the features in our model to identify the set of OTUs most predictive of infection status, resulting in 211 OTUs. Of the 211 OTUs, 38 were represented in the 41 overlapping OTUs obtained from our previous approaches. Using only the 41 overlapping OTUs as features in a second Random Forest model, we obtained 96% accuracy in predicting infection status.

### Classifying mallards using overlapping OTUs.

**FIG 4:**
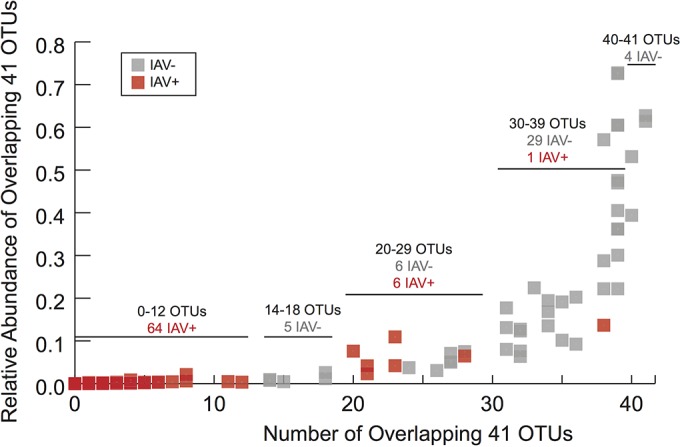
The number and abundance of the overlapping 41 OTUs in juvenile mallards.

Subsequently, we determined whether the overlapping 41 OTUs indicated the IAV condition for each sample. We found that the 41 overlapping OTUs were highly represented in IAV^−^ mallards and were not highly represented in IAV^+^ mallards (Fig. 3). IAV^−^ mallards had most of the 41 OTUs at high abundance, whereas IAV^+^ mallards frequently had few of the 41 OTUs and that those few OTUs occurred at low abundance (Fig. 4). More than 90% of the IAV^+^ mallards had 12 or fewer of the overlapping 41 OTUs, and 80% of the IAV^−^ mallards had more than 24 of the overlapping OTUs.

## DISCUSSION

This study was the first characterization of the cloacal microbiome of wild, free-ranging mallards. Anatomically, the cloaca differs from the gastrointestinal tract in that it interacts with the digestive system, the urogenital system, and the outside world (including sexual partners). Only a few sequence-based studies have been conducted on the cloacal microbiome of birds. As we found for the mallards, black-legged kittiwakes (*Rissa tridactyla*), another natural host for IAV (30), contain *Actinobacteria*, *Firmicutes*, and *Proteobacteria* in the cloacal microbiome and even share many genera with our data set (e.g., *Rothia*, *Streptococcus*, *Corynebacterium*, and *Enterococcus*) (31). The most abundant bacterial phyla reported in the cloacal microbiota of the barn swallow (*Hirundo rustica*), a passerine bird, were *Actinobacteria*, *Firmicutes*, *Proteobacteria*, *Bacteroidetes*, and *Tenericutes* (32). Several genera frequently found in our samples (e.g., *Rothia*, *Streptococcus*, *Corynebacterium*, *Enterococcus*, *Streptococcus*, and *Staphylococcus*) were detected in barn swallows as well.

All of the mallards sampled in this study were apparently healthy at the time that they were sampled, and a number of studies have shown that infection with low-pathogenicity IAVs does not cause gross or microscopic lesions in the gastrointestinal tract of waterfowl (4, 5, 6, 33). Thus, we assume that the microbiome effects were caused by the IAV and were not due to lower fitness in infected birds. All mallards were in their hatch year (likely <6 months of age) and were sampled on their breeding grounds prior to migration. Thus, our findings almost certainly reflect their first exposure to the particular virus strain isolated from their feces and may reflect their first exposure to any IAV. In an experimental infection study of IAV in mallards, Jourdain et al. (5) found that most viral shedding occurs during the first 7 days of infection. Despite the apparent lack of clinical disease or detectable pathology, some authors have suggested that infection may cause reduced fitness, perhaps due to IAV-induced decreases in nutrient uptake. For example, Latorre-Margalef et al. (17) found that body mass was significantly lower in free-ranging infected mallards than in uninfected mallards; however, prior infection status did not affect the speed or distance of subsequent migration. Importantly, we found that IAV^+^ mallards exhibited large differences in the composition of their cloacal microbiome, suggesting that IAV infection, and associated changes in the host microbiome, may have effects on the normal function of the gastrointestinal tract and perhaps of other organ systems and physiological responses (i.e., immune system responses) as well (4, 62, 63, 64). Although our study focused on a single species and a single anatomic location, our results indicate that future studies of host-IAV interactions may be incomplete if they do not include members of the microbiome as key players in the progress and outcome of infection.

Whether the observed differences in bacterial community composition associated with IAV infection in mallards represent a transient change in the cloacal microbiome is unknown. Although we assume that our IAV^−^ and IAV^+^ mallards had similar microbiomes prior to IAV infection, additional studies are needed to follow the microbiome composition before, during, and after infection. If IAV infection caused our observed differences in microbiome composition, an interesting future direction would be to verify that cloacal diversity returns to preinfection (IAV^−^) levels after recovery. Alternatively, mallards with more-diverse cloacal bacterial communities may be less susceptible to infection by IAVs, and the observed differences in bacterial community structure may instead reflect variations in the host susceptibility to infection rather than being a consequence of infection. Furthermore, if reduced diversity in the cloacal microbiome persists following infection, mallards exposed to IAVs may be more vulnerable to opportunistic infections. However, no studies have suggested that prior exposure, reflected by the presence of anti-IAV antibodies, is associated with decreased fitness or enhanced susceptibility to opportunistic infections.

More-diverse ecological communities are often more stable in response to perturbations because decreases in the abundances of some species are counterbalanced by increases in the abundances of others (34). Studies of macroorganisms (see, e.g., references 35 and 36) have found that greater species diversity is correlated with greater surface cover, which may help prevent the establishment of invasive species. Interactions between taxa may occur on surfaces in animal microbiomes, and, in some cases, low diversity may make hosts vulnerable to invasion. Still, it is important to recognize that diversity is not a panacea and that the consequences of diversity depend on complex interactions between host susceptibility and traits associated with invading species (37). In the human microbiome, diseases that incite a strong inflammatory response are known to cause changes (both decreases and increases) in bacterial OTU richness and diversity (reviewed in reference 38). For example, bacterial diversity in the lung is increased in patients with cystic fibrosis (39) and in patients with bacterial vaginosis (40). High microbial diversity is expected to occur in a healthy gut microbiome and in patients with Crohn’s disease (41), and patients with inflammatory bowel disease tend to have reduced bacterial diversity in their gastrointestinal tracts (42). However, it is unclear whether reduced diversity is a symptom or a cause of these conditions.

In this study, we used network modeling to identify a set of highly diagnostic cooccurring OTUs that would not have been detected by standard diversity analysis or differential-abundance-based statistics. Difference networks for the two IAV conditions revealed that D^−^ was a denser network, which was consistent with the observation that the IAV^−^ mallards had greater OTU diversity, richness, and evenness. Combining all three approaches revealed that a relatively small number of OTUs were significantly different across both the abundance and presence/absence patterns and that these OTUs contributed to differences associated with infection status, including infections by representatives of *Streptococcus* spp., *Veillonella dispar*, *Rothia mucilaginosa*, and *Prevotella*, among others.

Nearly all OTUs identified as contributing significantly to the observed differences were enriched in uninfected individuals. The only exception was a single OTU in the family *Micrococcaceae* (phylum *Actinobacteria*) that was enriched in IAV^+^ mallards (new reference OTU 462). In contrast, enrichment of specific bacterial taxa is associated with various medical conditions in humans and other animals. For example, enrichment by *Streptococcus* spp. was detected in human patients with irritable bowel disease (IBD) (reviewed in reference 43) and in chickens infected with pathogenic *Campylobacter jejuni* (44).

**FIG 5:**
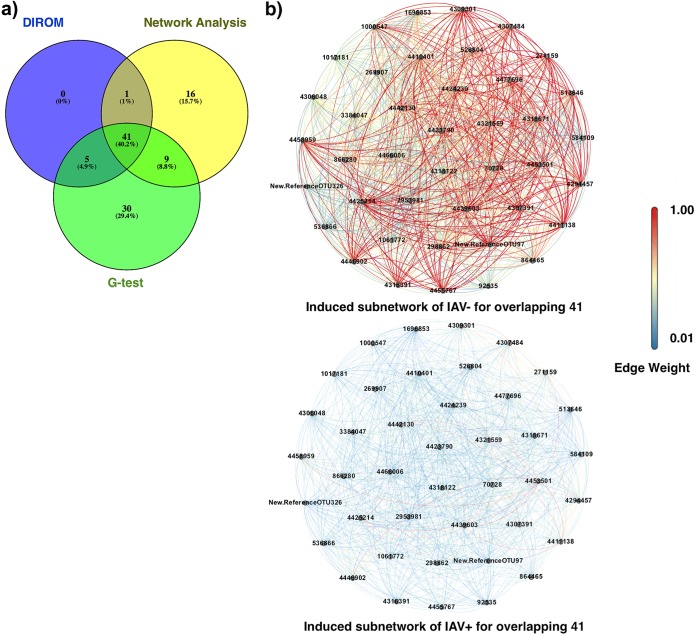
(a) Venn diagram showing the degree of overlap of the following different approaches: G-test for significant differences between groups (with Bonferroni corrections for false-discovery rate), DIROM, and unique network clusters. Unique networks consist of the set of OTUs that were obtained by clustering in D^+^ or D^−^ but not in both. (b) Induced subnetwork of IAV^+^ and IAV^−^ for the overlapping 41 OTUs. Both subnetworks are shown as roughly complete graphs (i.e., there exists an edge within every pair of OTUs); however, the edge weights in IAV^−^ are all of high value (average weighted degree of 25.4) and thus form a robust cluster showing strong cooccurrence patterns among the nodes, whereas in IAV^+^, edge weights among 41 overlapping OTUs were on average very low (average weighted degree of 1.7).

For an OTU to show up as significant in the G-test, the DIROM, and network clusters, it would need to be differentially enriched, differentially occurring, and highly cooccurring with other OTUs. OTUs identified as significant by all three methods did not fully overlap (Fig. 5a). This is because these methods detect different contributions of variance to the data. The network analysis identifies clusters based on the frequency of OTU cooccurrence within group, whereas the G-test and DIROM identify OTUs based on differential levels of enrichment between groups. Enrichment level and cooccurrence may be independent effects of IAV infection, in which case we would not expect to see a full overlap of the two analyses. However, both enrichment and cooccurrence contribute to intersample variance; thus, it was not unexpected for the OTUs identified by G-test and OTUs from the network analysis to overlap the highly variant OTUs without overlapping each other. However, the overlapping 41 OTUs were identified as significant in the three different approaches (Fig. 5 and Table 1). The intersection of the three methods contained OTU representatives of *Streptococcus*, *Rothia mucilaginosa*, *Veillonella dispar*, *Prevotella*, *Haemophilus*, and *Mycoplasma* and an unidentified OTU from the *Lactobacillales* order. These OTUs were assigned to these taxonomic classifications in QIIME, and these assignments were verified using BLAST. Interestingly, *Streptococcus*, *Haemophilus*, and *Veillonella* form consortia in the human oral microbiome and *Rothia* and *Prevotella* are common members as well (45). It would be interesting to investigate whether these taxa facilitate transmission of avian influenza virus to humans.

In conclusion, our methods revealed large differences in the compositions of the bacterial cloacal microbiomes of juvenile free-ranging mallards associated with IAV. Furthermore, we identified a set of significantly enriched, differentially occurring, and highly cooccurring OTUs that may be potential biomarker candidates for predicting infection status. Intersection of our analysis methods identified a concise subset of OTUs that best described the effect of IAV in mallards. This subset of OTUs provides specific targets for more-detailed studies addressing functional validation of in-depth interactions between individual taxa and IAVs. Experimental studies, with appropriate infected and noninfected controls sampled before, during, and after infection, are needed to confirm our findings and demonstrate causal relationships. Those studies should be performed with different IAV subtypes to explore the observed significant effect that different surface glycoproteins (HA and NA) have on bacterial diversity. Finally, such studies are also essential for assessing the relevance and ubiquity of our putative biomarker OTUs.

## MATERIALS AND METHODS

### Field collection and influenza testing.

Cloacal swabs were collected from 122 mallards for influenza testing and microbiome analysis. In the field, swabs were placed in separate vials containing 2 ml of ice-cold virus transport medium (VTM; medium 199 with Earle’s salts, l-glutamine, and sodium bicarbonate, plus 2 mU/liter penicillin G, 200 mg/liter streptomycin, 2 mU/liter polymyxin B, 250 mg/liter gentamicin, 0.5 mU/liter nystatin, 60 mg/liter ofloxacin, 200 mg/liter sulfamethoxazole, and 0.5% bovine serum albumin V). The samples were transported on ice to the laboratory, where they were stored at −80°C. Samples underwent one or more freeze/thaw cycles as aliquots were removed for influenza testing prior to microbiome analysis.

Samples were screened for the influenza matrix gene by reverse transcription-PCR (RT-PCR), viruses were isolated from matrix-positive samples by egg inoculation, and full-genome sequences were generated as described in reference 65. Known negative samples did not yield virus on egg inoculation and were matrix RT-PCR negative. We focused on juvenile mallards in this study in order to minimize differences that might arise in the composition of the microbiome in different age classes. More detail on these samples is included in Table S4 in the supplemental material).

10.1128/mSystems.00188-16.5 TABLE S4  Metadata file. Download TABLE S4, DOCX file, 0.03 MB. Copyright © 2017 Ganz et al. 2017 Ganz et al. This content is distributed under the terms of the Creative Commons Attribution 4.0 International license.

### DNA extraction.

DNA was extracted from cloacal swabs using a PowerSoil 96-well soil DNA isolation kit (MoBio, Carlsbad, CA). Samples in 96-well plates were incubated at 65°C for 10 min after addition of C1 solution. Each 96-well plate was then subjected to vortex mixing for 3-min using a plate shaker on the high setting (2,600 rpm), and then the standard kit protocol was followed. After elution, DNA was quantified using Qubit fluorometic quantitation (Invitrogen, South San Francisco, CA).

### Nested PCR to amplify and sequence the 16S rRNA gene.

DNA was characterized for bacterial diversity based on the V4 region of 16S rRNA gene following the methods of Caporaso et al. (46). When possible, 1.0 to 5.0 μl of template DNA was used for PCR. However, due to low DNA concentrations for some samples, bacterial DNA was amplified by a two-step PCR enrichment using primers 27F-YM+3 and 1492R to target V1 to V4 of the 16S rRNA gene (47). The 7-fold-degenerate primer 27f-YM+3 is composed of four parts 27f-YM (AGAGTTTGATYMTGGCTCAG) plus one part each of primers specific for the amplification of *Bifidobacteriaceae*, *Borrelia*, and *Chlamydiales* sequences (47). For the second PCR, the primers used were the bacterial/archaeal primers 515F/806R (46) modified by addition of an Illumina adaptor and an in-house barcode system (described in reference 48). After amplification, magnetic beads (Agencourt AMPure XP; Beckman Coulter, Inc., Indianapolis, IN) were used to clean the PCRs. Amplicons were quantified and characterized using Qubit fluorometic quantitation, quantitative PCR (qPCR), and a Bioanalyzer (Agilent Technologies, Santa Clara, CA) prior to sequencing. Libraries were sequenced using an Illumina MiSeq system, generating 250-bp paired-end amplicon reads. The amplicon data were multiplexed using dual-barcode combinations for each sample.

### Data analysis.

We used a custom script (available in a GitHub repository https://github.com/gjospin/scripts/blob/master/Demul_trim_prep.pl) to assign each pair of reads to their respective samples when parsing the raw data. This script allows for a 1-bp difference per barcode. The paired reads were then aligned and a consensus was computed using FLASH (49) with a maximum overlap of 120 and a minimum overlap of 70 (other parameters were left as the default). The custom script automatically demultiplexes the data into fastq files, executes FLASH, and parses its results to reformat the sequences with appropriate naming conventions for Quantitative Insights in Microbial Ecology (QIIME v.1.9.1; 46) in fasta format. Each sample was characterized for taxonomic composition (number and abundance) using QIIME. For presence/absence analyses, representative operational taxonomic units (OTUs) were clustered at the >97% identity level and an OTU table was constructed using QIIME’s pick_otus_through_otu_table.py script. Samples in the OTU table were rarefied to 5,000 reads per sample. After rarefaction, the total number of samples was 115 individuals: 71 mallards were IAV positive and 44 were IAV negative (Table S4).

We compared alpha diversity (mean species diversity per habitat) using the Shannon index as implemented in the *vegan* library (50) in R (51). We compared levels of OTU richness (numbers of OTUs found in each sample) and Pielou’s evenness (calculated by dividing the value for the Shannon index for diversity by the log of the value for OTU richness). We tested for statistical significance in alpha diversity measures using the Welch two-sample *t* test in R. We compared levels of beta diversity (the ratio between regional species diversity and local species diversity) using Bray-Curtis dissimilarity, and we used principal-coordinate analysis (PCoA) for ordination and clustering. We then used *Adonis*, a multivariate ANOVA based on dissimilarities, to test for significant categorical differences with 1,000 permutations in the *picante* library (52) in R.

### Identification of significant OTUs.

To identify the OTUs most responsible for the differentiation between IAV^−^ and IAV^+^ birds, we employed several strategies. The first and simplest method involved simply computing the difference in the relative occurrence metric (DIROM) results for each OTU in the IAV^−^ and IAV^+^ groups. For example, if an OTU was found in 40 of the 44 (0.91) IAV^−^ birds and 7 of the 71 (0.09) IAV^+^ birds, then that OTU had a DIROM value of 0.82 (the absolute difference between 0.91 and 0.09). We then sorted the OTUs in descending DIROM order, causing the most differentially occurring OTUs to be highest on the list. Next, we used the G-test to identify differences in OTU abundance between the different sample groups using the group_significance.py script in QIIME and the Bonferroni method to correct for multiple comparisons. We used Venny 2.1 (53) to examine the intersection of the results from the methods described above with the results of the network analysis described below. Finally, we used the Random Forest classifier (available in Python’s scikit-learn package; 29) with 3-fold cross-validation to learn the linear and nonlinear relationships among the features (here, relative OTU abundances) in order to find the set of the OTUs that are the most predictive of infection status.

### Building the IAV networks.

To identify OTUs that cooccur within an infection group (namely, IAV^+^ or IAV^−^), we used network analysis. Recent studies have shown that network inference techniques are useful for deciphering microbial relationships from cooccurrence patterns (18, 54, 55). Here, we built a network of the OTUs based on the presence/absence patterns across all samples. The goal was to see if there are clusters of OTUs that are more likely to cooccur in (only) one of the infection groups. We compared every pair of OTUs in one group to the same pair of OTUs in the other infection group, determining the cooccurrence patterns between them. If the cooccurrence patterns of a pair of OTUs differed by only a little between the infection groups, we ignored that pair of OTUs. Alternatively, if the patterns were very different, we studied that pair in more detail. We assumed that clusters of OTUs highly cooccurring in one group but not the other were infection dependent. We discuss the details of how we obtained these clusters below.

After identifying clustering of IAV^−^ and IAV^+^ mallards using ordination methods and ANOVA, we performed network analysis of the two groups as follows. First, we filtered the OTU table to remove taxa present in fewer than two individuals per IAV condition. Next, we binarized the OTU abundance matrix for presence/absence of OTUs. We define each sample as a binary presence/absence vector of OTUs, *x*_*j*_ϵ*X*, where *j* = (sample_*1*_, … sample_*N*_). Each vector value was multiplied by its transpose to obtain cooccurrence between all pairs of OTUs, and then the results were averaged across samples, for each IAV condition, to obtain the cooccurrence score for all pairs of OTUs as follows:

$$G=\frac{\sum_{j=1}^{N}X_{j}X_{j}^{T}}{N}$$

We explored the cooccurrence patterns in each group by building a network of OTUs where each OTU represented a node in the network and where there existed an edge between every two nodes. The elements of *G* are the scores for determining edge weights between two corresponding OTUs. In this study, we focused only on the positive cooccurrence patterns since only the positive patterns have the transitive property. We then visualized the networks of infected and uninfected groups in Fig. 2 using Gephi (56).

### Building the difference networks.

We then analyzed bacterial communities in IAV^+^ and IAV^−^ mallards by building separate difference networks (for each condition) based on the original networks (Fig. 2). Difference networks were calculated as the difference of edge weights between the two groups. Let us assume that we have OTU_*i*_ and OTU_*j*_ with an edge between them with weight *W*_IAV+_ in the IAV^+^ group. The same pair of OTUs, namely, (OTU_*i*_,OTU_*j*_), also occurs in the IAV^−^ group, with weight *W*_IAV−_. We built two difference networks, D^+^ and D^−^, and added OTU_*i*_ and OTU_*j*_ to the difference networks as nodes. If *W*_IAV+_ minus *W*_IAV−_ was positive and higher than a threshold ε value (0.2 was arbitrarily chosen throughout this study), this indicated that (OTU_*i*_,OTU_*j*_) had a strong cooccurrence pattern in the IAV^+^ group. We then added an edge between the terms (OTU_*i*_,OTU_*j*_) with weight *W*_IAV+_ to the difference network D^+^. Similarly, if *W*_IAV−_ minus *W*_IAV+_ was higher than ε, we added an edge between the terms (OTU_*i*_,OTU_*j*_) with weight *W*_IAV−_ to D^−^.

We denote a weighted graph *G* by *G* = (*V*, *E*) in which *V* is the set of vertices (nodes) and *E* is the set of edges between the vertices that connects them (|*V*| = *n*) with the weight function *W* : *E* → [0, 1]. Graph *G* should be undirected and irreflexive. We define our networks as the set of vertices *V* = (OTU_0_,OTU_1_,…OTU_*i*_,…OTU_*j*_,…OTU_*n*_). The network of the infected mallards (IAV^+^) is defined as follows:

$$G_{+}=(V,E_{+})\;\left[\text{and for simplicity, we call}W_{E_{+}}\left({\text{OTU}}_{i,}{\text{OTU}}_{j}\right)=W_{+ij}\right]$$

The network of the uninfected mallards (IAV^−^) is defined as follows:

$$G_{-}=(V,E_{-})\;\left[\text{and for simplicity, we call}\;W_{E_{-}}\;\left({\text{OTU}}_{i,}{\text{OTU}}_{j}\right)=W_{-ij}\right]$$

We can now define our difference networks (*G*_D+_ and *G*_D−_). The network of the D^+^ mallards is defined as follows:

$$G_{\text{D}+}=(V,E_{\text{D}+})\;$$

$$W_{E_{\text{D}+}}({\text{OTU}}_{i,}{\text{OTU}}_{j})=\left\{W_{+ij}|\left(W_{+ij}-W_{-ij}\right)>\text{ε and}\forall ({\text{OTU}}_{i},{\text{OTU}}_{j}),\text{where}\;{\text{OTU}}_{i}\;\in \;V\;\text{and}\;{\text{OTU}}_{j}\;\in \;V\;\text{and}\;i\neq j\right\}$$

And the network of the D^−^ mallards is defined as follows:

$$G_{\text{D}-}=(V,E_{\text{D}-})\;$$

$$W_{E_{\text{D}-}}({\text{OTU}}_{i,}{\text{OTU}}_{j})=\left\{W_{-ij}|\left(W_{-ij}-W_{+ij}\right)>\varepsilon \;\text{and}\forall ({\text{OTU}}_{i},{\text{OTU}}_{j}),\text{where}\;{\text{OTU}}_{i}\;\in \;V\;\text{and}\;{\text{OTU}}_{j}\;\in \;V\;\text{and}\;i\neq j\right\}$$

Once the difference networks D^+^ and D^−^ were constructed, we applied a threshold of 0.5 to remove the edges with low edge weights (Fig. 2). Then, we extracted the clusters (i.e., the groups of nodes that were more densely connected to each other than to the rest of the network) (26). We filtered out the clusters that were of size 3 or less (including singletons). The resulting clusters in the D^+^ and D^−^ networks contained 27 OTUs and 80 OTUs, respectively. We then examined which of the OTUs identified through the network analysis contributed the most to the differences between the IAV^+^ and IAV^−^ groups. Network analyses were performed in Python using the NetworkX package and Gephi (56, 57). The effect of removal of OTUs identified as significant by all the methods described above on PCoA clusters was determined (see Text S1 and Fig. S1 and S2 in the supplemental material).

10.1128/mSystems.00188-16.6 TEXT S1  Materials and Methods for testing for effects of OTU removal on ordination plots to accompany Fig. S1 and S2. Download TEXT S1, DOCX file, 0.1 MB. Copyright © 2017 Ganz et al. 2017 Ganz et al. This content is distributed under the terms of the Creative Commons Attribution 4.0 International license.

10.1128/mSystems.00188-16.7 FIG S2  PCoA plots of IAV^+^ and IAV^−^ mallards. (a) A total of 674 OTUs found in >2 individuals per group. (b) A total of 674 OTUs minus 7 OTUs that were unique in the IAV^+^ network. (c) A total of 674 OTUs minus 60 OTUs that were unique in the IAV^−^ network. (d) A total of 674 OTUs minus 20 mutual OTUs significant to both networks. (e) A total of 674 OTUs minus the 41 OTUs identified as overlapping from multiple approaches. (f) PCoA plots of IAV^+^ and IAV^−^ mallards calculated on the overlapping 41 OTUs only. Download FIG S2, PDF file, 0.3 MB. Copyright © 2017 Ganz et al. 2017 Ganz et al. This content is distributed under the terms of the Creative Commons Attribution 4.0 International license.

### Accession number(s).

The sequence data reported in this study have been submitted to the NCBI SRA database under BioProject accession number PRJNA358103.
