# Supplementary figures and images for: Community-Level Differences in the Microbiome of Healthy Wild Mallards and Those Infected by Influenza A Viruses
Source: mSystems. 2017 Feb 28;2(1):e00188-16. doi: 10.1128/mSystems.00188-16 (PMC5347185; doi:10.1128/mSystems.00188-16)

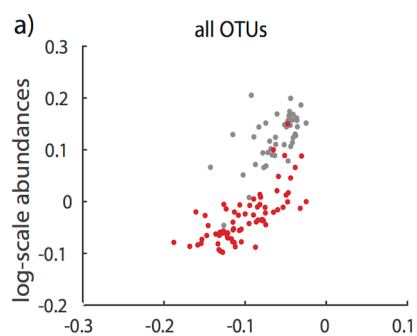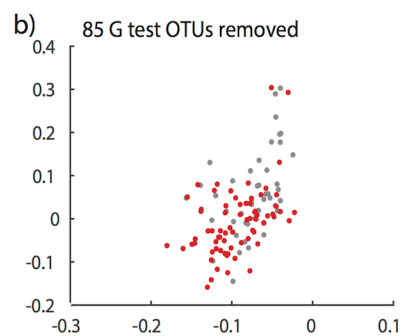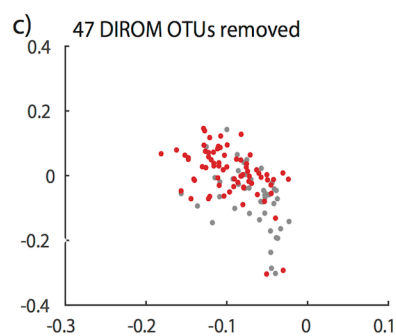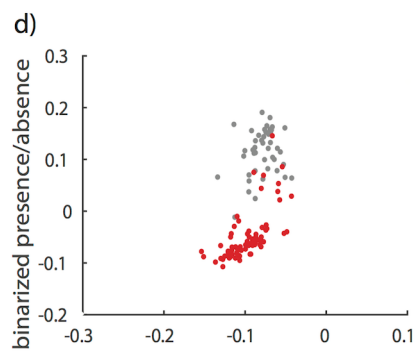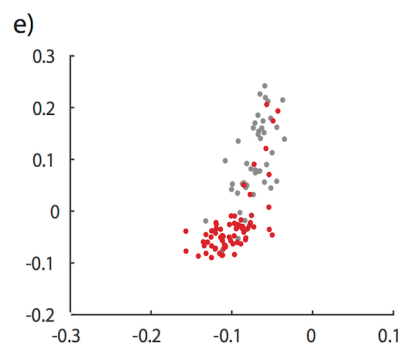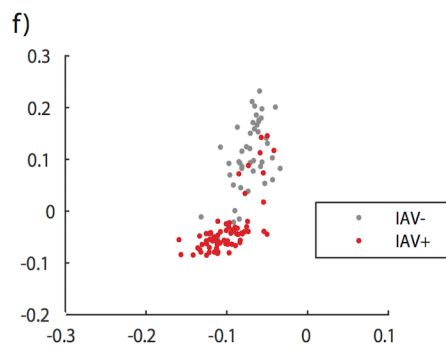

Supplement: FIG S1 [file sys001172081sf2.pdf]

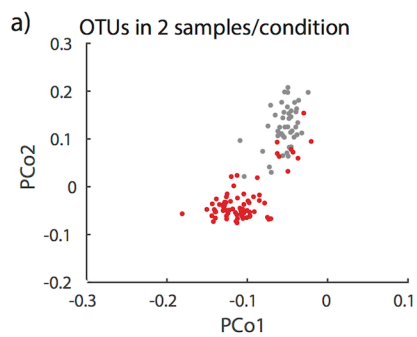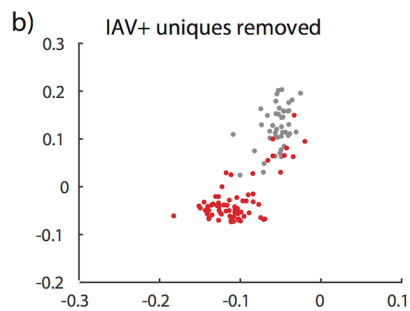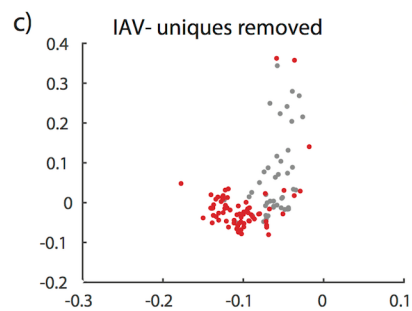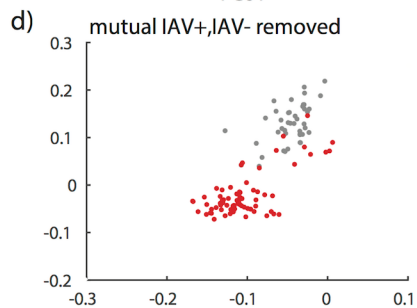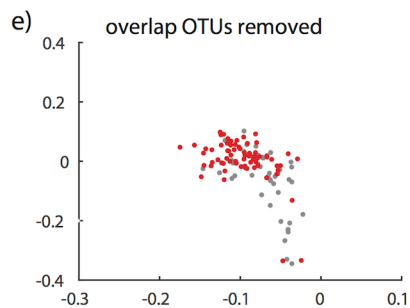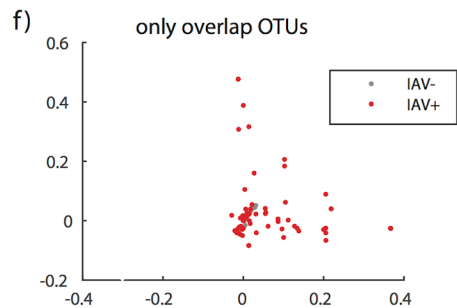

Supplement: FIG S2 [file sys001172081sf3.pdf]
