# Supplementary material for: Community-Level Differences in the Microbiome of Healthy Wild Mallards and Those Infected by Influenza A Viruses
Source: mSystems. 2017 Feb 28;2(1):e00188-16. doi: 10.1128/mSystems.00188-16 (PMC5347185; doi:10.1128/mSystems.00188-16)
Supplement: TABLE S1 [file sys001172081st4.docx]

Table S1. OTUs that differed by more than 50% in relative occurrence between IAV+ and IAV- mallards (difference in relative occurrence metric, DIROM). New.Reference is abbreviated as “NR”.

| \| **OTU** \| **DIROM score** \| **Phylum** \| **Class** \| **Order** \| **Family** \| **Genus** \| **Species** \| \| --- \| --- \| --- \| --- \| --- \| --- \| --- \| --- \| \| NR.OTU97 \| 0.92 \| Tenericutes \| Mollicutes \| Mycoplasmatales \| Mycoplasmataceae \| *Mycoplasma* \| \| \| 4455767 \| 0.83 \| Firmicutes \| Bacilli \| Lactobacillales \| Streptococcaceae \| *Streptococcus* \| \| \| 4477696 \| 0.80 \| Proteobacteria \| Gammaproteobacteria \| Pasteurellales \| Pasteurellaceae \| *Haemophilus* \| \| \| 271159 \| 0.80 \| Firmicutes \| Bacilli \| Lactobacillales \| \|  \|  \| \| 4309301 \| 0.79 \| Firmicutes \| Bacilli \| Lactobacillales \| Streptococcaceae \| *Streptococcus* \| \| \| 4307391 \| 0.79 \| Bacteroidetes \| Bacteroidia \| Bacteroidales \| Prevotellaceae \| *Prevotella* \| *melaninogenica* \| \| 4424239 \| 0.78 \| Firmicutes \| Bacilli \| Lactobacillales \| Streptococcaceae \| *Streptococcus* \| \| \| 4442130 \| 0.78 \| Firmicutes \| Bacilli \| Lactobacillales \| Streptococcaceae \| *Streptococcus* \| \| \| 4425214 \| 0.77 \| Firmicutes \| Bacilli \| Lactobacillales \| Streptococcaceae \| *Streptococcus* \| \| \| 513646 \| 0.77 \| Firmicutes \| Bacilli \| Lactobacillales \| Streptococcaceae \| *Streptococcus* \| \| \| 4318671 \| 0.76 \| Firmicutes \| Clostridia \| Clostridiales \| Veillonellaceae \| *Veillonella* \| *dispar* \| \| 4307484 \| 0.73 \| Firmicutes \| Bacilli \| Lactobacillales \| Streptococcaceae \| *Streptococcus* \| \| \| 526804 \| 0.73 \| Firmicutes \| Bacilli \| Lactobacillales \| Streptococcaceae \| *Streptococcus* \| \| \| 4316391 \| 0.73 \| Firmicutes \| Clostridia \| Clostridiales \| Veillonellaceae \| *Veillonella* \| *dispar* \| \| 4411138 \| 0.72 \| Actinobacteria \| Actinobacteria \| Actinomycetales \| Micrococcaceae \| *Rothia* \| *mucilaginosa* \| \| 1000547 \| 0.72 \| Firmicutes \| Bacilli \| Lactobacillales \| Streptococcaceae \| *Streptococcus* \| \| \| 4446902 \| 0.71 \| Firmicutes \| Bacilli \| Gemellales \| Gemellaceae \| \|  \| \| 4294457 \| 0.70 \| Actinobacteria \| Actinobacteria \| Actinomycetales \| Micrococcaceae \| *Rothia* \| *mucilaginosa* \| \| 4321559 \| 0.70 \| Bacteroidetes \| Bacteroidia \| Bacteroidales \| Porphyromonadaceae \| *Porphyromonas* \| \| \| 4410401 \| 0.70 \| Firmicutes \| Clostridia \| Clostridiales \| Veillonellaceae \| *Veillonella* \| *dispar* \| \| 4439603 \| 0.69 \| Firmicutes \| Bacilli \| Lactobacillales \| Streptococcaceae \| *Streptococcus* \| \| \| 866280 \| 0.69 \| Actinobacteria \| Actinobacteria \| Actinomycetales \| Micrococcaceae \| *Rothia* \| *mucilaginosa* \| \| 1696853 \| 0.68 \| Firmicutes \| Bacilli \| Lactobacillales \| \|  \|  \| \| 298862 \| 0.67 \| Firmicutes \| Bacilli \| Lactobacillales \| Streptococcaceae \| *Streptococcus* \| \| \| 4458959 \| 0.67 \| Firmicutes \| Clostridia \| Clostridiales \| Veillonellaceae \| *Veillonella* \| *parvula* \| \| 864465 \| 0.66 \| Firmicutes \| Bacilli \| Lactobacillales \| Streptococcaceae \| *Streptococcus* \| \| \| 4423790 \| 0.65 \| Bacteroidetes \| Bacteroidia \| Bacteroidales \| Porphyromonadaceae \| *Porphyromonas* \| *endodontalis* \| \| 70728 \| 0.65 \| Proteobacteria \| Gammaproteobacteria \| Pasteurellales \| Pasteurellaceae \| *Aggregatibacter* \| *pneumotropica* \| \| 2613485 \| 0.65 \| Bacteroidetes \| Bacteroidia \| Bacteroidales \| Porphyromonadaceae \| *Porphyromonas* \| \| \| 905211 \| 0.62 \| Actinobacteria \| Actinobacteria \| Actinomycetales \| Micrococcaceae \| *Rothia* \| *mucilaginosa* \| \| NR.OTU594 \| 0.60 \| Firmicutes \| Clostridia \| Clostridiales \| Veillonellaceae \| *Veillonella* \| *dispar* \| \| 1017181 \| 0.59 \| Actinobacteria \| Actinobacteria \| Actinomycetales \| Micrococcaceae \| *Rothia* \| *mucilaginosa* \| \| 4466006 \| 0.59 \| Actinobacteria \| Actinobacteria \| Actinomycetales \| Micrococcaceae \| *Rothia* \| *dentocariosa* \| \| 4318122 \| 0.59 \| Proteobacteria \| Gammaproteobacteria \| Pasteurellales \| Pasteurellaceae \| *Actinobacillus* \| *porcinus* \| \| 4306048 \| 0.59 \| Firmicutes \| Bacilli \| Lactobacillales \| Streptococcaceae \| *Streptococcus* \| \| \| 92535 \| 0.57 \| Firmicutes \| Bacilli \| Lactobacillales \| Streptococcaceae \| *Streptococcus* \| \| \| NR.OTU326 \| 0.56 \| Firmicutes \| Bacilli \| Lactobacillales \| Streptococcaceae \| *Streptococcus* \| \| \| 3384047 \| 0.56 \| Firmicutes \| Bacilli \| Lactobacillales \| Streptococcaceae \| *Streptococcus* \| \| \| 516611 \| 0.55 \| Firmicutes \| Bacilli \| Lactobacillales \| Streptococcaceae \| *Streptococcus* \| \| \| 269907 \| 0.55 \| Bacteroidetes \| Bacteroidia \| Bacteroidales \| [Paraprevotellaceae] \| *[Prevotella]* \|  \| \| 1061772 \| 0.53 \| Firmicutes \| Bacilli \|  \|  \|  \|  \| \| 584109 \| 0.53 \| Firmicutes \| Bacilli \| Lactobacillales \| Streptococcaceae \| *Streptococcus* \| \| \| 2953981 \| 0.52 \| Firmicutes \| Bacilli \| Lactobacillales \| Streptococcaceae \| *Streptococcus* \| \| \| 536866 \| 0.51 \| Firmicutes \| Bacilli \| Lactobacillales \| Streptococcaceae \| *Streptococcus* \| \| \| 70512 \| 0.51 \| Proteobacteria \| Gammaproteobacteria \| Pasteurellales \| Pasteurellaceae \| *Actinobacillus* \| *porcinus* \| \| 567427 \| 0.51 \| Firmicutes \| Bacilli \| Lactobacillales \| Streptococcaceae \| *Streptococcus* \| \| \| 4453501 \| 0.51 \| Firmicutes \| Clostridia \| Clostridiales \| Veillonellaceae \| *Veillonella* \| *dispar* \| |
| --- | --- | --- | --- | --- | --- | --- | --- | --- | --- | --- | --- | --- | --- | --- | --- | --- | --- | --- | --- | --- | --- | --- | --- | --- | --- | --- | --- | --- | --- | --- | --- | --- | --- | --- | --- | --- | --- | --- | --- | --- | --- | --- | --- | --- | --- | --- | --- | --- | --- | --- | --- | --- | --- | --- | --- | --- | --- | --- | --- | --- | --- | --- | --- | --- | --- | --- | --- | --- | --- | --- | --- | --- | --- | --- | --- | --- | --- | --- | --- | --- | --- | --- | --- | --- | --- | --- | --- | --- | --- | --- | --- | --- | --- | --- | --- | --- | --- | --- | --- | --- | --- | --- | --- | --- | --- | --- | --- | --- | --- | --- | --- | --- | --- | --- | --- | --- | --- | --- | --- | --- | --- | --- | --- | --- | --- | --- | --- | --- | --- | --- | --- | --- | --- | --- | --- | --- | --- | --- | --- | --- | --- | --- | --- | --- | --- | --- | --- | --- | --- | --- | --- | --- | --- | --- | --- | --- | --- | --- | --- | --- | --- | --- | --- | --- | --- | --- | --- | --- | --- | --- | --- | --- | --- | --- | --- | --- | --- | --- | --- | --- | --- | --- | --- | --- | --- | --- | --- | --- | --- | --- | --- | --- | --- | --- | --- | --- | --- | --- | --- | --- | --- | --- | --- | --- | --- | --- | --- | --- | --- | --- | --- | --- | --- | --- | --- | --- | --- | --- | --- | --- | --- | --- | --- | --- | --- | --- | --- | --- | --- | --- | --- | --- | --- | --- | --- | --- | --- | --- | --- | --- | --- | --- | --- | --- | --- | --- | --- | --- | --- | --- | --- | --- | --- | --- | --- | --- | --- | --- | --- | --- | --- | --- | --- | --- | --- | --- | --- | --- | --- | --- | --- | --- | --- | --- | --- | --- | --- | --- | --- | --- | --- | --- | --- | --- | --- | --- | --- | --- | --- | --- | --- | --- | --- | --- | --- | --- | --- | --- | --- | --- | --- | --- | --- | --- | --- | --- | --- | --- | --- | --- | --- | --- | --- | --- | --- | --- | --- | --- | --- | --- | --- | --- | --- | --- | --- | --- | --- | --- | --- | --- | --- | --- | --- | --- | --- | --- | --- | --- | --- | --- | --- | --- | --- | --- | --- | --- | --- | --- | --- | --- | --- | --- | --- | --- | --- | --- | --- | --- | --- | --- | --- | --- | --- | --- | --- | --- | --- | --- | --- | --- | --- | --- | --- | --- | --- | --- | --- | --- | --- | --- | --- | --- | --- | --- |
