# Supplementary material for: Community-Level Differences in the Microbiome of Healthy Wild Mallards and Those Infected by Influenza A Viruses
Source: mSystems. 2017 Feb 28;2(1):e00188-16. doi: 10.1128/mSystems.00188-16 (PMC5347185; doi:10.1128/mSystems.00188-16)
Supplement: TABLE S2 [file sys001172081st5.docx]

Table S2. Eight-five OTUs differed significantly in mean abundance between IAV+ and IAV− mallards based on the G-test (*P*-value corrected for multiple comparisons with Bonferroni method). New.Reference is abbreviated as “NR” and New.CleanUp.Reference is abbreviated as “NCR”.

| \| **OTU ID** \| **Test-statistic** \| ***P*-value** \| **IAV-** \| **IAV+** \| **Phylum** \| **Class** \| **Order** \| **Family** \| **Genus** \| **Species** \| \| --- \| --- \| --- \| --- \| --- \| --- \| --- \| --- \| --- \| --- \| --- \| \| 12574 \| 31.52 \| 0.0002 \| 6.18 \| 0.17 \| Actinobacteria \| Actinobacteria \| Actinomycetales \| Actinomycetaceae \| *Actinomyces* \|  \| \| 4466006 \| 39.94 \| 0.0000 \| 4.89 \| 0.37 \| Actinobacteria \| Actinobacteria \| Actinomycetales \| Micrococcaceae \| *Rothia* \| *dentocariosa* \| \| 4411138 \| 83.18 \| 0.0000 \| 127.2 \| 1.96 \| Actinobacteria \| Actinobacteria \| Actinomycetales \| Micrococcaceae \| *Rothia* \| *mucilaginosa* \| \| 866280 \| 67.21 \| 0.0000 \| 5.30 \| 0.07 \| Actinobacteria \| Actinobacteria \| Actinomycetales \| Micrococcaceae \| *Rothia* \| *mucilaginosa* \| \| 4294457 \| 61.35 \| 0.0000 \| 19.18 \| 1.20 \| Actinobacteria \| Actinobacteria \| Actinomycetales \| Micrococcaceae \| *Rothia* \| *mucilaginosa* \| \| 1017181 \| 48.15 \| 0.0000 \| 2.16 \| 0.08 \| Actinobacteria \| Actinobacteria \| Actinomycetales \| Micrococcaceae \| *Rothia* \| *mucilaginosa* \| \| 905211 \| 41.90 \| 0.0000 \| 1.55 \| 0.03 \| Actinobacteria \| Actinobacteria \| Actinomycetales \| Micrococcaceae \| *Rothia* \| *mucilaginosa* \| \| 886264 \| 32.90 \| 0.0001 \| 2.00 \| 0.01 \| Actinobacteria \| Actinobacteria \| Actinomycetales \| Micrococcaceae \| *Rothia* \| *mucilaginosa* \| \| NCR.OTU235199 \| 31.68 \| 0.0002 \| 0.70 \| 0.00 \| Actinobacteria \| Actinobacteria \| Actinomycetales \| Micrococcaceae \| *Rothia* \| *mucilaginosa* \| \| NR.OTU462 \| 21.62 \| 0.0341 \| 12.77 \| 64.90 \| Actinobacteria \| Actinobacteria \| Actinomycetales \| Micrococcaceae \|  \|  \| \| 4451251 \| 34.77 \| 0.0000 \| 2.43 \| 0.03 \| Actinobacteria \| Coriobacteriia \| Coriobacteriales \| Coriobacteriaceae \| *Atopobium* \|  \| \| 269907 \| 42.47 \| 0.0000 \| 8.98 \| 0.68 \| Bacteroidetes \| Bacteroidia \| Bacteroidales \| [Paraprevotellaceae] \| *[Prevotella]* \|  \| \| 4423790 \| 65.22 \| 0.0000 \| 8.02 \| 0.42 \| Bacteroidetes \| Bacteroidia \| Bacteroidales \| Porphyromonadaceae \| *Porphyromonas* \| *endodontalis* \| \| 4321559 \| 54.73 \| 0.0000 \| 13.84 \| 1.92 \| Bacteroidetes \| Bacteroidia \| Bacteroidales \| Porphyromonadaceae \| *Porphyromonas* \|  \| \| 2613485 \| 38.41 \| 0.0000 \| 3.68 \| 0.18 \| Bacteroidetes \| Bacteroidia \| Bacteroidales \| Porphyromonadaceae \| *Porphyromonas* \|  \| \| 4307391 \| 84.05 \| 0.0000 \| 102.8 \| 1.55 \| Bacteroidetes \| Bacteroidia \| Bacteroidales \| Prevotellaceae \| *Prevotella* \| *melaninogenica* \| \| 935742 \| 25.24 \| 0.0052 \| 5.16 \| 0.56 \| Bacteroidetes \| Bacteroidia \| Bacteroidales \| Prevotellaceae \| *Prevotella* \| *melaninogenica* \| \| 530206 \| 23.35 \| 0.0139 \| 0.61 \| 0.00 \| Bacteroidetes \| Bacteroidia \| Bacteroidales \| Prevotellaceae \| *Prevotella* \| *melaninogenica* \| \| 4460404 \| 21.74 \| 0.0319 \| 6.34 \| 0.55 \| Bacteroidetes \| Bacteroidia \| Bacteroidales \| Prevotellaceae \| *Prevotella* \| *melaninogenica* \| \| 4338372 \| 24.38 \| 0.0081 \| 5.50 \| 0.31 \| Bacteroidetes \| Bacteroidia \| Bacteroidales \| Prevotellaceae \| *Prevotella* \|  \| \| 4418243 \| 23.55 \| 0.0125 \| 10.98 \| 0.10 \| Bacteroidetes \| Bacteroidia \| Bacteroidales \| Prevotellaceae \| *Prevotella* \|  \| \| 4446902 \| 46.69 \| 0.0000 \| 12.36 \| 1.83 \| Firmicutes \| Bacilli \| Gemellales \| Gemellaceae \|  \|  \| \| 898207 \| 25.40 \| 0.0048 \| 0.48 \| 0.00 \| Firmicutes \| Bacilli \| Lactobacillales \| Carnobacteriaceae \| *Granulicatella* \|  \| \| 4339160 \| 23.27 \| 0.0145 \| 2.82 \| 0.85 \| Firmicutes \| Bacilli \| Lactobacillales \| Enterococcaceae \| *Vagococcus* \|  \| \| 4442130 \| 88.52 \| 0.0000 \| 91.14 \| 1.52 \| Firmicutes \| Bacilli \| Lactobacillales \| Streptococcaceae \| *Streptococcus* \|  \| \| 4439603 \| 79.01 \| 0.0000 \| 154.6 \| 4.38 \| Firmicutes \| Bacilli \| Lactobacillales \| Streptococcaceae \| *Streptococcus* \|  \| \| 4425214 \| 78.60 \| 0.0000 \| 51.41 \| 2.03 \| Firmicutes \| Bacilli \| Lactobacillales \| Streptococcaceae \| *Streptococcus* \|  \| \| 4309301 \| 73.73 \| 0.0000 \| 39.36 \| 1.37 \| Firmicutes \| Bacilli \| Lactobacillales \| Streptococcaceae \| *Streptococcus* \|  \| \| 4424239 \| 73.59 \| 0.0000 \| 6.84 \| 0.31 \| Firmicutes \| Bacilli \| Lactobacillales \| Streptococcaceae \| *Streptococcus* \|  \| \| 526804 \| 72.69 \| 0.0000 \| 8.80 \| 0.15 \| Firmicutes \| Bacilli \| Lactobacillales \| Streptococcaceae \| *Streptococcus* \|  \| \| 4455767 \| 71.50 \| 0.0000 \| 14.11 \| 0.46 \| Firmicutes \| Bacilli \| Lactobacillales \| Streptococcaceae \| *Streptococcus* \|  \| \| 4307484 \| 66.40 \| 0.0000 \| 6.50 \| 0.24 \| Firmicutes \| Bacilli \| Lactobacillales \| Streptococcaceae \| *Streptococcus* \|  \| \| 864465 \| 62.14 \| 0.0000 \| 2.80 \| 0.13 \| Firmicutes \| Bacilli \| Lactobacillales \| Streptococcaceae \| *Streptococcus* \|  \| \| 4306048 \| 55.61 \| 0.0000 \| 4.18 \| 0.13 \| Firmicutes \| Bacilli \| Lactobacillales \| Streptococcaceae \| *Streptococcus* \|  \| \| 92535 \| 55.17 \| 0.0000 \| 1.84 \| 0.00 \| Firmicutes \| Bacilli \| Lactobacillales \| Streptococcaceae \| *Streptococcus* \|  \| \| 1000547 \| 53.65 \| 0.0000 \| 6.84 \| 0.38 \| Firmicutes \| Bacilli \| Lactobacillales \| Streptococcaceae \| *Streptococcus* \|  \| \| 513646 \| 52.50 \| 0.0000 \| 5.00 \| 0.01 \| Firmicutes \| Bacilli \| Lactobacillales \| Streptococcaceae \| *Streptococcus* \|  \| \| 516611 \| 52.19 \| 0.0000 \| 1.34 \| 0.01 \| Firmicutes \| Bacilli \| Lactobacillales \| Streptococcaceae \| *Streptococcus* \|  \| \| 567427 \| 49.82 \| 0.0000 \| 4.41 \| 0.01 \| Firmicutes \| Bacilli \| Lactobacillales \| Streptococcaceae \| *Streptococcus* \|  \| \| 584109 \| 49.26 \| 0.0000 \| 2.00 \| 0.03 \| Firmicutes \| Bacilli \| Lactobacillales \| Streptococcaceae \| *Streptococcus* \|  \| \| 2953981 \| 46.84 \| 0.0000 \| 3.36 \| 0.03 \| Firmicutes \| Bacilli \| Lactobacillales \| Streptococcaceae \| *Streptococcus* \|  \| \| 298862 \| 43.55 \| 0.0000 \| 8.84 \| 1.69 \| Firmicutes \| Bacilli \| Lactobacillales \| Streptococcaceae \| *Streptococcus* \|  \| \| 863124 \| 37.43 \| 0.0000 \| 1.34 \| 0.01 \| Firmicutes \| Bacilli \| Lactobacillales \| Streptococcaceae \| *Streptococcus* \|  \| \| 4396519 \| 36.81 \| 0.0000 \| 3.16 \| 0.32 \| Firmicutes \| Bacilli \| Lactobacillales \| Streptococcaceae \| *Streptococcus* \|  \| \| NR.OTU326 \| 35.63 \| 0.0000 \| 1.50 \| 0.14 \| Firmicutes \| Bacilli \| Lactobacillales \| Streptococcaceae \| *Streptococcus* \|  \| \| 536866 \| 34.91 \| 0.0000 \| 1.18 \| 0.10 \| Firmicutes \| Bacilli \| Lactobacillales \| Streptococcaceae \| *Streptococcus* \|  \| \| 3384047 \| 33.38 \| 0.0001 \| 4.66 \| 0.37 \| Firmicutes \| Bacilli \| Lactobacillales \| Streptococcaceae \| *Streptococcus* \|  \| \| 4302049 \| 32.77 \| 0.0001 \| 1.18 \| 0.01 \| Firmicutes \| Bacilli \| Lactobacillales \| Streptococcaceae \| *Streptococcus* \|  \| \| 4315974 \| 31.82 \| 0.0002 \| 1.89 \| 0.04 \| Firmicutes \| Bacilli \| Lactobacillales \| Streptococcaceae \| *Streptococcus* \|  \| \| NR.OTU106 \| 25.47 \| 0.0046 \| 0.39 \| 0.00 \| Firmicutes \| Bacilli \| Lactobacillales \| Streptococcaceae \| *Streptococcus* \|  \| \| NCR.OTU270648 \| 25.37 \| 0.0048 \| 0.66 \| 0.00 \| Firmicutes \| Bacilli \| Lactobacillales \| Streptococcaceae \| *Streptococcus* \|  \| \| 517754 \| 23.37 \| 0.0137 \| 1.18 \| 0.00 \| Firmicutes \| Bacilli \| Lactobacillales \| Streptococcaceae \| *Streptococcus* \|  \| \| NCR.OTU174319 \| 23.35 \| 0.0138 \| 0.61 \| 0.00 \| Firmicutes \| Bacilli \| Lactobacillales \| Streptococcaceae \| *Streptococcus* \|  \| \| 4415177 \| 23.19 \| 0.0150 \| 0.95 \| 0.04 \| Firmicutes \| Bacilli \| Lactobacillales \| Streptococcaceae \| *Streptococcus* \|  \| \| 3449122 \| 21.37 \| 0.0388 \| 0.41 \| 0.00 \| Firmicutes \| Bacilli \| Lactobacillales \| Streptococcaceae \| *Streptococcus* \|  \| \| 271159 \| 78.52 \| 0.0000 \| 34.25 \| 0.87 \| Firmicutes \| Bacilli \| Lactobacillales \|  \|  \|  \| \| 1696853 \| 55.49 \| 0.0000 \| 2.25 \| 0.07 \| Firmicutes \| Bacilli \| Lactobacillales \|  \|  \|  \| \| 1061772 \| 37.26 \| 0.0000 \| 1.52 \| 0.06 \| Firmicutes \| Bacilli \|  \|  \|  \|  \| \| 4404577 \| 28.43 \| 0.0010 \| 3.75 \| 0.32 \| Firmicutes \| Clostridia \| Clostridiales \| Peptostreptococcaceae \| *Peptostreptococcus* \|  \| \| 4453501 \| 74.92 \| 0.0000 \| 315.0 \| 7.66 \| Firmicutes \| Clostridia \| Clostridiales \| Veillonellaceae \| *Veillonella* \| *dispar* \| \| 4410401 \| 67.88 \| 0.0000 \| 4.52 \| 0.11 \| Firmicutes \| Clostridia \| Clostridiales \| Veillonellaceae \| *Veillonella* \| *dispar* \| \| 4318671 \| 58.57 \| 0.0000 \| 13.75 \| 0.39 \| Firmicutes \| Clostridia \| Clostridiales \| Veillonellaceae \| *Veillonella* \| *dispar* \| \| 4316391 \| 57.61 \| 0.0000 \| 5.61 \| 0.14 \| Firmicutes \| Clostridia \| Clostridiales \| Veillonellaceae \| *Veillonella* \| *dispar* \| \| NR.OTU594 \| 49.67 \| 0.0000 \| 2.32 \| 0.03 \| Firmicutes \| Clostridia \| Clostridiales \| Veillonellaceae \| *Veillonella* \| *dispar* \| \| 181155 \| 30.07 \| 0.0004 \| 1.23 \| 0.03 \| Firmicutes \| Clostridia \| Clostridiales \| Veillonellaceae \| *Veillonella* \| *dispar* \| \| NR.OTU330 \| 26.44 \| 0.0028 \| 0.89 \| 0.01 \| Firmicutes \| Clostridia \| Clostridiales \| Veillonellaceae \| *Veillonella* \| *dispar* \| \| 4334770 \| 23.39 \| 0.0136 \| 0.36 \| 0.00 \| Firmicutes \| Clostridia \| Clostridiales \| Veillonellaceae \| *Veillonella* \| *dispar* \| \| 4458306 \| 23.36 \| 0.0138 \| 0.48 \| 0.00 \| Firmicutes \| Clostridia \| Clostridiales \| Veillonellaceae \| *Veillonella* \| *dispar* \| \| NCR.OTU252119 \| 22.09 \| 0.0267 \| 0.50 \| 0.01 \| Firmicutes \| Clostridia \| Clostridiales \| Veillonellaceae \| *Veillonella* \| *dispar* \| \| 4458959 \| 37.44 \| 0.0000 \| 23.57 \| 1.99 \| Firmicutes \| Clostridia \| Clostridiales \| Veillonellaceae \| *Veillonella* \| *parvula* \| \| 4422456 \| 25.08 \| 0.0056 \| 9.16 \| 0.87 \| Firmicutes \| Clostridia \| Clostridiales \| Veillonellaceae \| *Veillonella* \| *parvula* \| \| 3801267 \| 21.85 \| 0.0303 \| 3.43 \| 0.89 \| Firmicutes \| Clostridia \| Clostridiales \| Veillonellaceae \| *Veillonella* \| *parvula* \| \| NR.OTU2 \| 33.18 \| 0.0001 \| 10.43 \| 1.45 \| Firmicutes \| Clostridia \| Clostridiales \| Veillonellaceae \| *Veillonella* \|  \| \| 4319899 \| 29.50 \| 0.0006 \| 5.34 \| 0.11 \| Fusobacteria \| Fusobacteriia \| Fusobacteriales \| Fusobacteriaceae \| *Fusobacterium* \|  \| \| 4452538 \| 21.54 \| 0.0356 \| 3.77 \| 0.04 \| Fusobacteria \| Fusobacteriia \| Fusobacteriales \| Fusobacteriaceae \| *Fusobacterium* \|  \| \| 4323555 \| 21.49 \| 0.0365 \| 3.52 \| 0.23 \| Fusobacteria \| Fusobacteriia \| Fusobacteriales \| Fusobacteriaceae \| *Fusobacterium* \|  \| \| 4318122 \| 42.36 \| 0.0000 \| 9.77 \| 2.68 \| Proteobacteria \| Gamma-proteobacteria \| Pasteurellales \| Pasteurellaceae \| *Actinobacillus* \| *porcinus* \| \| 4404220 \| 31.57 \| 0.0002 \| 4.52 \| 1.69 \| Proteobacteria \| Gamma-proteobacteria \| Pasteurellales \| Pasteurellaceae \| *Actinobacillus* \|  \| \| 70728 \| 45.69 \| 0.0000 \| 7.98 \| 2.15 \| Proteobacteria \| Gamma-proteobacteria \| Pasteurellales \| Pasteurellaceae \| *Aggregatibacter* \| *pneumotropica* \| \| 109413 \| 22.94 \| 0.0171 \| 1.14 \| 0.14 \| Proteobacteria \| Gamma-proteobacteria \| Pasteurellales \| Pasteurellaceae \| *Haemophilus* \| *parainfluenzae* \| \| 4477696 \| 71.46 \| 0.0000 \| 41.18 \| 1.08 \| Proteobacteria \| Gamma-proteobacteria \| Pasteurellales \| Pasteurellaceae \| *Haemophilus* \|  \| \| NR.OTU97 \| 84.94 \| 0.0000 \| 54.30 \| 1.10 \| Tenericutes \| Mollicutes \| Mycoplasmatales \| Mycoplasmataceae \| *Mycoplasma* \|  \| \| NR.OTU549 \| 28.02 \| 0.0012 \| 1.77 \| 0.03 \| Tenericutes \| Mollicutes \| Mycoplasmatales \| Mycoplasmataceae \| *Mycoplasma* \|  \| \| NCR.OTU243493 \| 27.47 \| 0.0016 \| 0.45 \| 0.00 \| Tenericutes \| Mollicutes \| Mycoplasmatales \| Mycoplasmataceae \| *Mycoplasma* \|  \| \| NR.OTU431 \| 25.19 \| 0.0053 \| 0.68 \| 0.15 \| Tenericutes \| Mollicutes \| Mycoplasmatales \| Mycoplasmataceae \| *Mycoplasma* \|  \| |
| --- | --- | --- | --- | --- | --- | --- | --- | --- | --- | --- | --- | --- | --- | --- | --- | --- | --- | --- | --- | --- | --- | --- | --- | --- | --- | --- | --- | --- | --- | --- | --- | --- | --- | --- | --- | --- | --- | --- | --- | --- | --- | --- | --- | --- | --- | --- | --- | --- | --- | --- | --- | --- | --- | --- | --- | --- | --- | --- | --- | --- | --- | --- | --- | --- | --- | --- | --- | --- | --- | --- | --- | --- | --- | --- | --- | --- | --- | --- | --- | --- | --- | --- | --- | --- | --- | --- | --- | --- | --- | --- | --- | --- | --- | --- | --- | --- | --- | --- | --- | --- | --- | --- | --- | --- | --- | --- | --- | --- | --- | --- | --- | --- | --- | --- | --- | --- | --- | --- | --- | --- | --- | --- | --- | --- | --- | --- | --- | --- | --- | --- | --- | --- | --- | --- | --- | --- | --- | --- | --- | --- | --- | --- | --- | --- | --- | --- | --- | --- | --- | --- | --- | --- | --- | --- | --- | --- | --- | --- | --- | --- | --- | --- | --- | --- | --- | --- | --- | --- | --- | --- | --- | --- | --- | --- | --- | --- | --- | --- | --- | --- | --- | --- | --- | --- | --- | --- | --- | --- | --- | --- | --- | --- | --- | --- | --- | --- | --- | --- | --- | --- | --- | --- | --- | --- | --- | --- | --- | --- | --- | --- | --- | --- | --- | --- | --- | --- | --- | --- | --- | --- | --- | --- | --- | --- | --- | --- | --- | --- | --- | --- | --- | --- | --- | --- | --- | --- | --- | --- | --- | --- | --- | --- | --- | --- | --- | --- | --- | --- | --- | --- | --- | --- | --- | --- | --- | --- | --- | --- | --- | --- | --- | --- | --- | --- | --- | --- | --- | --- | --- | --- | --- | --- | --- | --- | --- | --- | --- | --- | --- | --- | --- | --- | --- | --- | --- | --- | --- | --- | --- | --- | --- | --- | --- | --- | --- | --- | --- | --- | --- | --- | --- | --- | --- | --- | --- | --- | --- | --- | --- | --- | --- | --- | --- | --- | --- | --- | --- | --- | --- | --- | --- | --- | --- | --- | --- | --- | --- | --- | --- | --- | --- | --- | --- | --- | --- | --- | --- | --- | --- | --- | --- | --- | --- | --- | --- | --- | --- | --- | --- | --- | --- | --- | --- | --- | --- | --- | --- | --- | --- | --- | --- | --- | --- | --- | --- | --- | --- | --- | --- | --- | --- | --- | --- | --- | --- | --- | --- | --- | --- | --- | --- | --- | --- | --- | --- | --- | --- | --- | --- | --- | --- | --- | --- | --- | --- | --- | --- | --- | --- | --- | --- | --- | --- | --- | --- | --- | --- | --- | --- | --- | --- | --- | --- | --- | --- | --- | --- | --- | --- | --- | --- | --- | --- | --- | --- | --- | --- | --- | --- | --- | --- | --- | --- | --- | --- | --- | --- | --- | --- | --- | --- | --- | --- | --- | --- | --- | --- | --- | --- | --- | --- | --- | --- | --- | --- | --- | --- | --- | --- | --- | --- | --- | --- | --- | --- | --- | --- | --- | --- | --- | --- | --- | --- | --- | --- | --- | --- | --- | --- | --- | --- | --- | --- | --- | --- | --- | --- | --- | --- | --- | --- | --- | --- | --- | --- | --- | --- | --- | --- | --- | --- | --- | --- | --- | --- | --- | --- | --- | --- | --- | --- | --- | --- | --- | --- | --- | --- | --- | --- | --- | --- | --- | --- | --- | --- | --- | --- | --- | --- | --- | --- | --- | --- | --- | --- | --- | --- | --- | --- | --- | --- | --- | --- | --- | --- | --- | --- | --- | --- | --- | --- | --- | --- | --- | --- | --- | --- | --- | --- | --- | --- | --- | --- | --- | --- | --- | --- | --- | --- | --- | --- | --- | --- | --- | --- | --- | --- | --- | --- | --- | --- | --- | --- | --- | --- | --- | --- | --- | --- | --- | --- | --- | --- | --- | --- | --- | --- | --- | --- | --- | --- | --- | --- | --- | --- | --- | --- | --- | --- | --- | --- | --- | --- | --- | --- | --- | --- | --- | --- | --- | --- | --- | --- | --- | --- | --- | --- | --- | --- | --- | --- | --- | --- | --- | --- | --- | --- | --- | --- | --- | --- | --- | --- | --- | --- | --- | --- | --- | --- | --- | --- | --- | --- | --- | --- | --- | --- | --- | --- | --- | --- | --- | --- | --- | --- | --- | --- | --- | --- | --- | --- | --- | --- | --- | --- | --- | --- | --- | --- | --- | --- | --- | --- | --- | --- | --- | --- | --- | --- | --- | --- | --- | --- | --- | --- | --- | --- | --- | --- | --- | --- | --- | --- | --- | --- | --- | --- | --- | --- | --- | --- | --- | --- | --- | --- | --- | --- | --- | --- | --- | --- | --- | --- | --- | --- | --- | --- | --- | --- | --- | --- | --- | --- | --- | --- | --- | --- | --- | --- | --- | --- | --- | --- | --- | --- | --- | --- | --- | --- | --- | --- | --- | --- | --- | --- | --- | --- | --- | --- | --- | --- | --- | --- | --- | --- | --- | --- | --- | --- | --- | --- | --- | --- | --- | --- | --- | --- | --- | --- | --- | --- | --- | --- | --- | --- | --- | --- | --- | --- | --- | --- | --- | --- | --- | --- | --- | --- | --- | --- | --- | --- | --- | --- | --- | --- | --- | --- | --- | --- | --- | --- | --- | --- | --- | --- | --- | --- | --- | --- | --- | --- | --- | --- | --- | --- | --- | --- | --- | --- | --- | --- | --- | --- | --- | --- | --- | --- | --- | --- | --- | --- | --- | --- | --- | --- | --- | --- | --- | --- | --- | --- | --- | --- | --- | --- | --- | --- | --- | --- | --- | --- | --- | --- | --- | --- | --- | --- | --- | --- | --- | --- | --- | --- | --- | --- | --- | --- | --- | --- | --- | --- | --- | --- | --- | --- | --- | --- | --- | --- | --- | --- | --- | --- | --- | --- | --- | --- | --- | --- | --- | --- | --- | --- | --- | --- | --- | --- | --- | --- | --- | --- | --- | --- | --- | --- | --- | --- | --- | --- | --- | --- | --- | --- | --- | --- | --- | --- | --- | --- | --- | --- | --- | --- | --- | --- | --- | --- | --- | --- | --- | --- | --- | --- | --- | --- | --- |
