# Supplementary material for: Community-Level Differences in the Microbiome of Healthy Wild Mallards and Those Infected by Influenza A Viruses
Source: mSystems. 2017 Feb 28;2(1):e00188-16. doi: 10.1128/mSystems.00188-16 (PMC5347185; doi:10.1128/mSystems.00188-16)
Supplement: TABLE S3 [file sys001172081st6.docx]

Table S3. Taxonomic assignments for OTUs identified in network modeling as significantly clustered. New.Reference is abbreviated as “NR”.

| \| **Network** \| **OTU** \| **Domain** \| **Phylum** \| **Class** \| **Order** \| **Family** \| **Genus** \| **Species** \| \| --- \| --- \| --- \| --- \| --- \| --- \| --- \| --- \| --- \| \| IAV- \| 12574 \| Bacteria \| Actinobacteria \| Actinobacteria \| Actinomycetales \| Actinomycetaceae \| *Actinomyces* \| \| \| IAV- \| 70512 \| Bacteria \| Proteobacteria \| Gammaproteobacteria \| Pasteurellales \| Pasteurellaceae \| *Actinobacillus* \| *porcinus* \| \| IAV- \| 70728 \| Bacteria \| Proteobacteria \| Gammaproteobacteria \| Pasteurellales \| Pasteurellaceae \| *Aggregatibacter* \| *pneumotropica* \| \| IAV- \| 92535 \| Bacteria \| Firmicutes \| Bacilli \| Lactobacillales \| Streptococcaceae \| *Streptococcus* \| \| \| IAV- \| 208949 \| Bacteria \| Bacteroidetes \| Bacteroidia \| Bacteroidales \| Bacteroidaceae \| *Bacteroides* \|  \| \| IAV- \| 269907 \| Bacteria \| Bacteroidetes \| Bacteroidia \| Bacteroidales \| [Paraprevotellaceae] \| *[Prevotella]* \|  \| \| IAV- \| 271159 \| Bacteria \| Firmicutes \| Bacilli \| Lactobacillales \| \|  \|  \| \| IAV- \| 298862 \| Bacteria \| Firmicutes \| Bacilli \| Lactobacillales \| Streptococcaceae \| *Streptococcus* \| \| \| IAV- \| 513646 \| Bacteria \| Firmicutes \| Bacilli \| Lactobacillales \| Streptococcaceae \| *Streptococcus* \| \| \| IAV- \| 526804 \| Bacteria \| Firmicutes \| Bacilli \| Lactobacillales \| Streptococcaceae \| *Streptococcus* \| \| \| IAV- \| 536866 \| Bacteria \| Firmicutes \| Bacilli \| Lactobacillales \| Streptococcaceae \| *Streptococcus* \| \| \| IAV- \| 572889 \| Bacteria \| Fusobacteria \| Fusobacteriia \| Fusobacteriales \| Fusobacteriaceae \| *Fusobacterium* \| \| \| IAV- \| 584109 \| Bacteria \| Firmicutes \| Bacilli \| Lactobacillales \| Streptococcaceae \| *Streptococcus* \| \| \| IAV- \| 864465 \| Bacteria \| Firmicutes \| Bacilli \| Lactobacillales \| Streptococcaceae \| *Streptococcus* \| \| \| IAV- \| 866280 \| Bacteria \| Actinobacteria \| Actinobacteria \| Actinomycetales \| Micrococcaceae \| *Rothia* \| *mucilaginosa* \| \| IAV- \| 1000547 \| Bacteria \| Firmicutes \| Bacilli \| Lactobacillales \| Streptococcaceae \| *Streptococcus* \| \| \| IAV- \| 1017181 \| Bacteria \| Actinobacteria \| Actinobacteria \| Actinomycetales \| Micrococcaceae \| *Rothia* \| *mucilaginosa* \| \| IAV- \| 1061772 \| Bacteria \| Firmicutes \| Bacilli \|  \|  \|  \|  \| \| IAV- \| 1696853 \| Bacteria \| Firmicutes \| Bacilli \| Lactobacillales \| \|  \|  \| \| IAV- \| 2953981 \| Bacteria \| Firmicutes \| Bacilli \| Lactobacillales \| Streptococcaceae \| *Streptococcus* \| \| \| IAV- \| 3384047 \| Bacteria \| Firmicutes \| Bacilli \| Lactobacillales \| Streptococcaceae \| *Streptococcus* \| \| \| IAV- \| 3801267 \| Bacteria \| Firmicutes \| Clostridia \| Clostridiales \| Veillonellaceae \| *Veillonella* \| *parvula* \| \| IAV- \| 4045626 \| Bacteria \| Proteobacteria \| Gammaproteobacteria \| Oceanospirillales \| Halomonadaceae \| *Halomonas* \|  \| \| IAV- \| 4294457 \| Bacteria \| Actinobacteria \| Actinobacteria \| Actinomycetales \| Micrococcaceae \| *Rothia* \| *mucilaginosa* \| \| IAV- \| 4306048 \| Bacteria \| Firmicutes \| Bacilli \| Lactobacillales \| Streptococcaceae \| *Streptococcus* \| \| \| IAV- \| 4307391 \| Bacteria \| Bacteroidetes \| Bacteroidia \| Bacteroidales \| Prevotellaceae \| *Prevotella* \| *melaninogenica* \| \| IAV- \| 4307484 \| Bacteria \| Firmicutes \| Bacilli \| Lactobacillales \| Streptococcaceae \| *Streptococcus* \| \| \| IAV- \| 4309301 \| Bacteria \| Firmicutes \| Bacilli \| Lactobacillales \| Streptococcaceae \| *Streptococcus* \| \| \| IAV- \| 4316391 \| Bacteria \| Firmicutes \| Clostridia \| Clostridiales \| Veillonellaceae \| *Veillonella* \| *dispar* \| \| IAV- \| 4318122 \| Bacteria \| Proteobacteria \| Gammaproteobacteria \| Pasteurellales \| Pasteurellaceae \| *Actinobacillus* \| *porcinus* \| \| IAV- \| 4318671 \| Bacteria \| Firmicutes \| Clostridia \| Clostridiales \| Veillonellaceae \| *Veillonella* \| *dispar* \| \| IAV- \| 4321559 \| Bacteria \| Bacteroidetes \| Bacteroidia \| Bacteroidales \| Porphyromonadaceae \| *Porphyromonas* \| \| \| IAV- \| 4323555 \| Bacteria \| Fusobacteria \| Fusobacteriia \| Fusobacteriales \| Fusobacteriaceae \| *Fusobacterium* \| \| \| IAV- \| 4339160 \| Bacteria \| Firmicutes \| Bacilli \| Lactobacillales \| Enterococcaceae \| *Vagococcus* \|  \| \| IAV- \| 4345285 \| Bacteria \| Firmicutes \| Bacilli \| Bacillales \| Staphylococcaceae \| *Staphylococcus* \| \| \| IAV- \| 4388775 \| Bacteria \| Firmicutes \| Clostridia \| Clostridiales \| Veillonellaceae \| *Veillonella* \| *dispar* \| \| IAV- \| 4396519 \| Bacteria \| Firmicutes \| Bacilli \| Lactobacillales \| Streptococcaceae \| *Streptococcus* \| \| \| IAV- \| 4404220 \| Bacteria \| Proteobacteria \| Gammaproteobacteria \| Pasteurellales \| Pasteurellaceae \| *Actinobacillus* \| \| \| IAV- \| 4410401 \| Bacteria \| Firmicutes \| Clostridia \| Clostridiales \| Veillonellaceae \| *Veillonella* \| *dispar* \| \| IAV- \| 4411138 \| Bacteria \| Actinobacteria \| Actinobacteria \| Actinomycetales \| Micrococcaceae \| *Rothia* \| *mucilaginosa* \| \| IAV- \| 4422456 \| Bacteria \| Firmicutes \| Clostridia \| Clostridiales \| Veillonellaceae \| *Veillonella* \| *parvula* \| \| IAV- \| 4423790 \| Bacteria \| Bacteroidetes \| Bacteroidia \| Bacteroidales \| Porphyromonadaceae \| *Porphyromonas* \| *endodontalis* \| \| IAV- \| 4424239 \| Bacteria \| Firmicutes \| Bacilli \| Lactobacillales \| Streptococcaceae \| *Streptococcus* \| \| \| IAV- \| 4425214 \| Bacteria \| Firmicutes \| Bacilli \| Lactobacillales \| Streptococcaceae \| *Streptococcus* \| \| \| IAV- \| 4439603 \| Bacteria \| Firmicutes \| Bacilli \| Lactobacillales \| Streptococcaceae \| *Streptococcus* \| \| \| IAV- \| 4442130 \| Bacteria \| Firmicutes \| Bacilli \| Lactobacillales \| Streptococcaceae \| *Streptococcus* \| \| \| IAV- \| 4446902 \| Bacteria \| Firmicutes \| Bacilli \| Gemellales \| Gemellaceae \| \|  \| \| IAV- \| 4453501 \| Bacteria \| Firmicutes \| Clostridia \| Clostridiales \| Veillonellaceae \| *Veillonella* \| *dispar* \| \| IAV- \| 4455767 \| Bacteria \| Firmicutes \| Bacilli \| Lactobacillales \| Streptococcaceae \| *Streptococcus* \| \| \| IAV- \| 4458959 \| Bacteria \| Firmicutes \| Clostridia \| Clostridiales \| Veillonellaceae \| *Veillonella* \| *parvula* \| \| IAV- \| 4460404 \| Bacteria \| Bacteroidetes \| Bacteroidia \| Bacteroidales \| Prevotellaceae \| *Prevotella* \| *melaninogenica* \| \| IAV- \| 4466006 \| Bacteria \| Actinobacteria \| Actinobacteria \| Actinomycetales \| Micrococcaceae \| *Rothia* \| *dentocariosa* \| \| IAV- \| 4477696 \| Bacteria \| Proteobacteria \| Gammaproteobacteria \| Pasteurellales \| Pasteurellaceae \| *Haemophilus* \| \| \| IAV- \| NR.OTU2 \| Bacteria \| Firmicutes \| Clostridia \| Clostridiales \| Veillonellaceae \| *Veillonella* \|  \| \| IAV- \| NR.OTU326 \| Bacteria \| Firmicutes \| Bacilli \| Lactobacillales \| Streptococcaceae \| *Streptococcus* \| \| \| IAV- \| NR.OTU438 \| Unassigned \|  \|  \|  \|  \|  \|  \| \| IAV- \| NR.OTU477 \| Bacteria \| Fusobacteria \| Fusobacteriia \| Fusobacteriales \| Leptotrichiaceae \| \|  \| \| IAV- \| NR.OTU624 \| Bacteria \| Proteobacteria \| Epsilonproteobacteria \| Campylobacterales \| Campylobacteraceae \| *Campylobacter* \| \| \| IAV- \| NR.OTU81 \| Bacteria \| Fusobacteria \| Fusobacteriia \| Fusobacteriales \| Leptotrichiaceae \| \|  \| \| IAV- \| NR.OTU97 \| Bacteria \| Tenericutes \| Mollicutes \| Mycoplasmatales \| Mycoplasmataceae \| *Mycoplasma* \| \| \| IAV+ \| 225259 \| Bacteria \| Proteobacteria \| Betaproteobacteria \| Burkholderiales \| Comamonadaceae \| \|  \| \| IAV+ \| 238830 \| Bacteria \| Actinobacteria \| Actinobacteria \| Actinomycetales \| Actinomycetaceae \| *Arcanobacterium* \| \| \| IAV+ \| 862357 \| Bacteria \| Bacteroidetes \| Sphingobacteriia \| Sphingobacteriales \| Sphingobacteriaceae \| *Pedobacter* \|  \| \| IAV+ \| 1099710 \| Bacteria \| Firmicutes \| Clostridia \| Clostridiales \| Peptococcaceae \| *Peptococcus* \| \| \| IAV+ \| 4303213 \| Bacteria \| Firmicutes \| Clostridia \| Clostridiales \| [Tissierellaceae] \| *Parvimonas* \|  \| \| IAV+ \| 4354703 \| Bacteria \| Bacteroidetes \| Sphingobacteriia \| Sphingobacteriales \| Sphingobacteriaceae \| *Pedobacter* \|  \| \| IAV+ \| NR.OTU171 \| Bacteria \| Firmicutes \| Clostridia \| Clostridiales \| [Tissierellaceae] \| *Parvimonas* \|  \| \| IAV+/IAV- \| 226338 \| Bacteria \| Firmicutes \| Bacilli \| Lactobacillales \| Enterococcaceae \| *Enterococcus* \| *cecorum* \| \| IAV+/IAV- \| 227343 \| Bacteria \| Proteobacteria \| Gammaproteobacteria \| Xanthomonadales \| Xanthomonadaceae \| \|  \| \| IAV+/IAV- \| 269930 \| Bacteria \| Proteobacteria \| Gammaproteobacteria \| Pseudomonadales \| Pseudomonadaceae \| \|  \| \| IAV+/IAV- \| 284123 \| Bacteria \| Proteobacteria \| Epsilonproteobacteria \| Campylobacterales \| Campylobacteraceae \| *Campylobacter* \| \| \| IAV+/IAV- \| 557974 \| Bacteria \| Proteobacteria \| Gammaproteobacteria \| Pseudomonadales \| Pseudomonadaceae \| *Pseudomonas* \| \| \| IAV+/IAV- \| 815573 \| Bacteria \| Firmicutes \| Clostridia \| Clostridiales \| Lachnospiraceae \| *Coprococcus* \| \| \| IAV+/IAV- \| 851799 \| Bacteria \| Bacteroidetes \| Sphingobacteriia \| Sphingobacteriales \| Sphingobacteriaceae \| *Sphingobacterium* \| \| \| IAV+/IAV- \| 1122504 \| Bacteria \| Bacteroidetes \| Flavobacteriia \| Flavobacteriales \| Flavobacteriaceae \| \|  \| \| IAV+/IAV- \| 3494917 \| Bacteria \| Firmicutes \| Erysipelotrichi \| Erysipelotrichales \| Erysipelotrichaceae \| \|  \| \| IAV+/IAV- \| 4353264 \| Bacteria \| Proteobacteria \| Alphaproteobacteria \| Caulobacterales \| Caulobacteraceae \| \|  \| \| IAV+/IAV- \| 4439398 \| Bacteria \| Fusobacteria \| Fusobacteriia \| Fusobacteriales \| Fusobacteriaceae \| *Fusobacterium* \| \| \| IAV+/IAV- \| NR.OTU28 \| Bacteria \| Firmicutes \| Erysipelotrichi \| Erysipelotrichales \| Erysipelotrichaceae \| \|  \| \| IAV+/IAV- \| NR.OTU451 \| Bacteria \| Proteobacteria \| Epsilonproteobacteria \| Campylobacterales \| Campylobacteraceae \| *Campylobacter* \| \| \| IAV+/IAV- \| NR.OTU462 \| Bacteria \| Actinobacteria \| Actinobacteria \| Actinomycetales \| Micrococcaceae \| \|  \| \| IAV+/IAV- \| NR.OTU5 \| Bacteria \| Actinobacteria \| Actinobacteria \| Actinomycetales \| Actinomycetaceae \| *Varibaculum* \| \| \| IAV+/IAV- \| NR.OTU500 \| Unassigned \|  \|  \|  \|  \|  \|  \| \| IAV+/IAV- \| NR.OTU552 \| Bacteria \| Proteobacteria \| Gammaproteobacteria \| Pasteurellales \| Pasteurellaceae \| \|  \| \| IAV+/IAV- \| NR.OTU568 \| Bacteria \| Firmicutes \| Clostridia \| Clostridiales \| Veillonellaceae \| *Veillonella* \| *dispar* \| \| IAV+/IAV- \| NR.OTU701 \| Bacteria \| Firmicutes \| Clostridia \| Clostridiales \|  \|  \|  \| \| IAV+/IAV- \| NR.OTU712 \| Bacteria \| Bacteroidetes \| Bacteroidia \| Bacteroidales \| Porphyromonadaceae \| *Parabacteroides* \| \| |
| --- | --- | --- | --- | --- | --- | --- | --- | --- | --- | --- | --- | --- | --- | --- | --- | --- | --- | --- | --- | --- | --- | --- | --- | --- | --- | --- | --- | --- | --- | --- | --- | --- | --- | --- | --- | --- | --- | --- | --- | --- | --- | --- | --- | --- | --- | --- | --- | --- | --- | --- | --- | --- | --- | --- | --- | --- | --- | --- | --- | --- | --- | --- | --- | --- | --- | --- | --- | --- | --- | --- | --- | --- | --- | --- | --- | --- | --- | --- | --- | --- | --- | --- | --- | --- | --- | --- | --- | --- | --- | --- | --- | --- | --- | --- | --- | --- | --- | --- | --- | --- | --- | --- | --- | --- | --- | --- | --- | --- | --- | --- | --- | --- | --- | --- | --- | --- | --- | --- | --- | --- | --- | --- | --- | --- | --- | --- | --- | --- | --- | --- | --- | --- | --- | --- | --- | --- | --- | --- | --- | --- | --- | --- | --- | --- | --- | --- | --- | --- | --- | --- | --- | --- | --- | --- | --- | --- | --- | --- | --- | --- | --- | --- | --- | --- | --- | --- | --- | --- | --- | --- | --- | --- | --- | --- | --- | --- | --- | --- | --- | --- | --- | --- | --- | --- | --- | --- | --- | --- | --- | --- | --- | --- | --- | --- | --- | --- | --- | --- | --- | --- | --- | --- | --- | --- | --- | --- | --- | --- | --- | --- | --- | --- | --- | --- | --- | --- | --- | --- | --- | --- | --- | --- | --- | --- | --- | --- | --- | --- | --- | --- | --- | --- | --- | --- | --- | --- | --- | --- | --- | --- | --- | --- | --- | --- | --- | --- | --- | --- | --- | --- | --- | --- | --- | --- | --- | --- | --- | --- | --- | --- | --- | --- | --- | --- | --- | --- | --- | --- | --- | --- | --- | --- | --- | --- | --- | --- | --- | --- | --- | --- | --- | --- | --- | --- | --- | --- | --- | --- | --- | --- | --- | --- | --- | --- | --- | --- | --- | --- | --- | --- | --- | --- | --- | --- | --- | --- | --- | --- | --- | --- | --- | --- | --- | --- | --- | --- | --- | --- | --- | --- | --- | --- | --- | --- | --- | --- | --- | --- | --- | --- | --- | --- | --- | --- | --- | --- | --- | --- | --- | --- | --- | --- | --- | --- | --- | --- | --- | --- | --- | --- | --- | --- | --- | --- | --- | --- | --- | --- | --- | --- | --- | --- | --- | --- | --- | --- | --- | --- | --- | --- | --- | --- | --- | --- | --- | --- | --- | --- | --- | --- | --- | --- | --- | --- | --- | --- | --- | --- | --- | --- | --- | --- | --- | --- | --- | --- | --- | --- | --- | --- | --- | --- | --- | --- | --- | --- | --- | --- | --- | --- | --- | --- | --- | --- | --- | --- | --- | --- | --- | --- | --- | --- | --- | --- | --- | --- | --- | --- | --- | --- | --- | --- | --- | --- | --- | --- | --- | --- | --- | --- | --- | --- | --- | --- | --- | --- | --- | --- | --- | --- | --- | --- | --- | --- | --- | --- | --- | --- | --- | --- | --- | --- | --- | --- | --- | --- | --- | --- | --- | --- | --- | --- | --- | --- | --- | --- | --- | --- | --- | --- | --- | --- | --- | --- | --- | --- | --- | --- | --- | --- | --- | --- | --- | --- | --- | --- | --- | --- | --- | --- | --- | --- | --- | --- | --- | --- | --- | --- | --- | --- | --- | --- | --- | --- | --- | --- | --- | --- | --- | --- | --- | --- | --- | --- | --- | --- | --- | --- | --- | --- | --- | --- | --- | --- | --- | --- | --- | --- | --- | --- | --- | --- | --- | --- | --- | --- | --- | --- | --- | --- | --- | --- | --- | --- | --- | --- | --- | --- | --- | --- | --- | --- | --- | --- | --- | --- | --- | --- | --- | --- | --- | --- | --- | --- | --- | --- | --- | --- | --- | --- | --- | --- | --- | --- | --- | --- | --- | --- | --- | --- | --- | --- | --- | --- | --- | --- | --- | --- | --- | --- | --- | --- | --- | --- | --- | --- | --- | --- | --- | --- | --- | --- | --- | --- | --- | --- | --- | --- | --- | --- | --- | --- | --- | --- | --- | --- | --- | --- | --- | --- | --- | --- | --- | --- | --- | --- | --- | --- | --- | --- | --- | --- | --- | --- | --- | --- | --- | --- | --- | --- | --- | --- | --- | --- | --- | --- | --- | --- | --- | --- | --- | --- | --- | --- | --- | --- | --- | --- | --- | --- | --- | --- | --- | --- | --- | --- | --- | --- | --- | --- | --- | --- | --- | --- | --- | --- | --- | --- | --- | --- | --- | --- | --- | --- | --- | --- | --- | --- | --- | --- | --- | --- | --- | --- | --- | --- | --- | --- | --- | --- | --- | --- | --- | --- | --- | --- | --- | --- | --- | --- | --- | --- | --- | --- | --- | --- | --- | --- | --- | --- | --- | --- | --- | --- | --- | --- | --- | --- | --- | --- | --- | --- | --- | --- | --- | --- | --- | --- | --- | --- | --- | --- | --- | --- | --- | --- | --- | --- | --- | --- | --- | --- | --- | --- | --- | --- | --- | --- | --- | --- | --- | --- | --- | --- | --- | --- | --- | --- | --- | --- | --- | --- | --- | --- | --- | --- | --- | --- | --- | --- | --- | --- |
