# Supplementary material for: Community-Level Differences in the Microbiome of Healthy Wild Mallards and Those Infected by Influenza A Viruses
Source: mSystems. 2017 Feb 28;2(1):e00188-16. doi: 10.1128/mSystems.00188-16 (PMC5347185; doi:10.1128/mSystems.00188-16)
Supplement: TABLE S4 [file sys001172081st7.docx]

Table S4. Metadata file

| \| SampleID \| Influenza \| IAV_level \| Ct value \| Genotype \| HASubType \| NASubType \| HANASubType \| Sex \| Date \| Location \| Year \| Description \| \| --- \| --- \| --- \| --- \| --- \| --- \| --- \| --- \| --- \| --- \| --- \| --- \| --- \| \| UCD.09.05149 \| Pos \| 2 \| 35.49 \| 25 \| 5 \| 2 \| H5N2 \| F \| 25-Jul-09 \| SuisunMarsh/ArnoldRanch,SolanoCounty \| 2009 \| Pos_2009_Anasplatyrhynchos \| \| UCD.09.05174 \| Pos \| 3 \| 33.01 \| 25 \| 5 \| 2 \| H5N2 \| M \| 25-Jul-09 \| SuisunMarsh/Sunrise,SolanoCounty \| 2009 \| Pos_2009_Anasplatyrhynchos \| \| UCD.09.05191 \| Pos \| NA \| NA \| 25 \| 5 \| 2 \| H5N2 \| M \| 27-Jul-09 \| SuisunMarsh/ArnoldRanch,SolanoCounty \| 2009 \| Pos_2009_Anasplatyrhynchos \| \| UCD.09.05192 \| Pos \| 3 \| 31.93 \| 12 \| 4 \| 2 \| H4N2 \| M \| 27-Jul-09 \| SuisunMarsh/ArnoldRanch,SolanoCounty \| 2009 \| Pos_2009_Anasplatyrhynchos \| \| UCD.09.05197 \| Pos \| 1 \| 41.95 \| 25 \| 5 \| 2 \| H5N2 \| F \| 27-Jul-09 \| SuisunMarsh/ArnoldRanch,SolanoCounty \| 2009 \| Pos_2009_Anasplatyrhynchos \| \| UCD.09.05205 \| Pos \| 2 \| 35.41 \| NA \| 4 \| 2.6 \| H4N2.6 \| F \| 27-Jul-09 \| SuisunMarsh/ArnoldRanch,SolanoCounty \| 2009 \| Pos_2009_Anasplatyrhynchos \| \| UCD.09.05212 \| Pos \| 1 \| 43.54 \| 25 \| 5 \| 2 \| H5N2 \| F \| 27-Jul-09 \| SuisunMarsh/Sunrise,SolanoCounty \| 2009 \| Pos_2009_Anasplatyrhynchos \| \| UCD.09.05219 \| Pos \| 3 \| 33 \| 25 \| 5 \| 2 \| H5N2 \| M \| 27-Jul-09 \| SuisunMarsh/Sunrise,SolanoCounty \| 2009 \| Pos_2009_Anasplatyrhynchos \| \| UCD.09.05222 \| Pos \| 3 \| 33.43 \| 25 \| 5 \| 2 \| H5N2 \| M \| 27-Jul-09 \| SuisunMarsh/Sunrise,SolanoCounty \| 2009 \| Pos_2009_Anasplatyrhynchos \| \| UCD.09.05233 \| Neg \| 0 \| NA \| NA \| neg \| neg \| neg \| M \| 27-Jul-09 \| SuisunMarsh/Balboa,SolanoCounty \| 2009 \| Neg_2009_Anasplatyrhynchos \| \| UCD.09.05235 \| Neg \| 0 \| NA \| NA \| neg \| neg \| neg \| M \| 27-Jul-09 \| SuisunMarsh/Balboa,SolanoCounty \| 2009 \| Neg_2009_Anasplatyrhynchos \| \| UCD.09.05236 \| Neg \| 0 \| NA \| NA \| neg \| neg \| neg \| M \| 27-Jul-09 \| SuisunMarsh/Balboa,SolanoCounty \| 2009 \| Neg_2009_Anasplatyrhynchos \| \| UCD.09.05238 \| Neg \| 0 \| NA \| NA \| neg \| neg \| neg \| M \| 27-Jul-09 \| SuisunMarsh/Balboa,SolanoCounty \| 2009 \| Neg_2009_Anasplatyrhynchos \| \| UCD.09.05239 \| Neg \| 0 \| NA \| NA \| neg \| neg \| neg \| M \| 27-Jul-09 \| SuisunMarsh/Balboa,SolanoCounty \| 2009 \| Neg_2009_Anasplatyrhynchos \| \| UCD.09.05240 \| Neg \| 0 \| NA \| NA \| neg \| neg \| neg \| F \| 27-Jul-09 \| SuisunMarsh/Balboa,SolanoCounty \| 2009 \| Neg_2009_Anasplatyrhynchos \| \| UCD.09.05241 \| Neg \| 0 \| NA \| NA \| neg \| neg \| neg \| M \| 27-Jul-09 \| SuisunMarsh/Balboa,SolanoCounty \| 2009 \| Neg_2009_Anasplatyrhynchos \| \| UCD.09.05243 \| Neg \| 0 \| NA \| NA \| neg \| neg \| neg \| M \| 27-Jul-09 \| SuisunMarsh/Balboa,SolanoCounty \| 2009 \| Neg_2009_Anasplatyrhynchos \| \| UCD.09.05244 \| Neg \| 0 \| NA \| NA \| neg \| neg \| neg \| F \| 27-Jul-09 \| SuisunMarsh/Balboa,SolanoCounty \| 2009 \| Neg_2009_Anasplatyrhynchos \| \| UCD.09.05255.V \| Pos \| 1 \| 40.93 \| 5 \| 3 \| 8 \| H3N8 \| F \| 28-Jul-09 \| SuisunMarsh/ArnoldRanch,SolanoCounty \| 2009 \| Pos_2009_Anasplatyrhynchos \| \| UCD.09.05271 \| Pos \| 2 \| 35.13 \| 14 \| 4 \| 6 \| H4N6 \| M \| 29-Jul-09 \| SuisunMarsh/ArnoldRanch,SolanoCounty \| 2009 \| Pos_2009_Anasplatyrhynchos \| \| UCD.09.05282 \| Pos \| 3 \| 34.91 \| NA \| 3.4 \| 2.8 \| H3.4N2.8 \| M \| 29-Jul-09 \| SuisunMarsh/ArnoldRanch,SolanoCounty \| 2009 \| Pos_2009_Anasplatyrhynchos \| \| UCD.09.05296 \| Pos \| 3 \| 34.79 \| 25 \| 5 \| 2 \| H5N2 \| M \| 29-Jul-09 \| SuisunMarsh/Sunrise,SolanoCounty \| 2009 \| Pos_2009_Anasplatyrhynchos \| \| UCD.09.05319 \| Pos \| 2 \| 35.96 \| 25 \| 5 \| 2 \| H5N2 \| M \| 23-Jul-09 \| SuisunMarsh/ArnoldRanch,SolanoCounty \| 2009 \| Pos_2009_Anasplatyrhynchos \| \| UCD.09.05491 \| Pos \| 2 \| 36.78 \| 25 \| 5 \| 2 \| H5N2 \| M \| 23-Jul-09 \| SuisunMarsh/ArnoldRanch,SolanoCounty \| 2009 \| Pos_2009_Anasplatyrhynchos \| \| UCD.09.05495 \| Pos \| 2 \| 35.28 \| 13 \| 4 \| 2 \| H4N2 \| F \| 23-Jul-09 \| SuisunMarsh/ArnoldRanch,SolanoCounty \| 2009 \| Pos_2009_Anasplatyrhynchos \| \| UCD.09.05502 \| Pos \| 3 \| 32.63 \| 25 \| 5 \| 2 \| H5N2 \| M \| 23-Jul-09 \| SuisunMarsh/Balboa,SolanoCounty \| 2009 \| Pos_2009_Anasplatyrhynchos \| \| UCD.10.01139.CP \| Neg \| 0 \| NA \| NA \| neg \| neg \| neg \| F \| 27-Jul-10 \| GrizzlyIslandWildlifeArea(GIWA),SolanoCounty \| 2010 \| Neg_2010_Anasplatyrhynchos \| \| UCD.10.01140.CP \| Neg \| 0 \| NA \| NA \| neg \| neg \| neg \| M \| 27-Jul-10 \| GrizzlyIslandWildlifeArea(GIWA),SolanoCounty \| 2010 \| Neg_2010_Anasplatyrhynchos \| \| UCD.10.01141.CP \| Neg \| 0 \| NA \| NA \| neg \| neg \| neg \| F \| 27-Jul-10 \| GrizzlyIslandWildlifeArea(GIWA),SolanoCounty \| 2010 \| Neg_2010_Anasplatyrhynchos \| \| UCD.10.01142.CP \| Neg \| 0 \| NA \| NA \| neg \| neg \| neg \| F \| 27-Jul-10 \| GrizzlyIslandWildlifeArea(GIWA),SolanoCounty \| 2010 \| Neg_2010_Anasplatyrhynchos \| \| UCD.10.01143.CP \| Neg \| 0 \| NA \| NA \| neg \| neg \| neg \| F \| 27-Jul-10 \| GrizzlyIslandWildlifeArea(GIWA),SolanoCounty \| 2010 \| Neg_2010_Anasplatyrhynchos \| \| UCD.10.01144.CP \| Neg \| 0 \| NA \| NA \| neg \| neg \| neg \| F \| 27-Jul-10 \| GrizzlyIslandWildlifeArea(GIWA),SolanoCounty \| 2010 \| Neg_2010_Anasplatyrhynchos \| \| UCD.10.01145.CP \| Neg \| 0 \| NA \| NA \| neg \| neg \| neg \| F \| 27-Jul-10 \| GrizzlyIslandWildlifeArea(GIWA),SolanoCounty \| 2010 \| Neg_2010_Anasplatyrhynchos \| \| UCD.10.01146.CP \| Neg \| 0 \| NA \| NA \| neg \| neg \| neg \| M \| 27-Jul-10 \| GrizzlyIslandWildlifeArea(GIWA),SolanoCounty \| 2010 \| Neg_2010_Anasplatyrhynchos \| \| UCD.10.01147.CP \| Neg \| 0 \| NA \| NA \| neg \| neg \| neg \| M \| 27-Jul-10 \| GrizzlyIslandWildlifeArea(GIWA),SolanoCounty \| 2010 \| Neg_2010_Anasplatyrhynchos \| \| UCD.10.01148.CP \| Neg \| 0 \| NA \| NA \| neg \| neg \| neg \| F \| 27-Jul-10 \| GrizzlyIslandWildlifeArea(GIWA),SolanoCounty \| 2010 \| Neg_2010_Anasplatyrhynchos \| \| UCD.10.01156.CP \| Pos \| 3 \| 32.3 \| 15 \| 4 \| 6 \| H4N6 \| M \| 27-Jul-10 \| GrizzlyIslandWildlifeArea(GIWA),SolanoCounty \| 2010 \| Pos_2010_Anasplatyrhynchos \| \| UCD.10.1188.CV \| Pos \| 2 \| 35.84 \| 16 \| 4 \| 6 \| H4N6 \| F \| 29-Jul-10 \| GrizzlyIslandWildlifeArea(GIWA),SolanoCounty \| 2010 \| Pos_2010_Anasplatyrhynchos \| \| UCD.10.1297.CP \| Pos \| 2 \| 35.5 \| 16 \| 4 \| 6 \| H4N6 \| F \| 30-Jul-10 \| GrizzlyIslandWildlifeArea(GIWA),SolanoCounty \| 2010 \| Pos_2010_Anasplatyrhynchos \| \| UCD.11.02521 \| Pos \| 3 \| 32.09 \| 6 \| 3 \| 8 \| H3N8 \| F \| 12-Aug-11 \| GrizzlyIslandWildlifeArea(GIWA),SolanoCounty \| 2011 \| Pos_2011_Anasplatyrhynchos \| \| UCD.11.02522 \| Neg \| 0 \| NA \| NA \| neg \| neg \| neg \| F \| 12-Aug-11 \| GrizzlyIslandWildlifeArea(GIWA),SolanoCounty \| 2011 \| Neg_2011_Anasplatyrhynchos \| \| UCD.11.02524 \| Neg \| 0 \| NA \| NA \| neg \| neg \| neg \| M \| 12-Aug-11 \| GrizzlyIslandWildlifeArea(GIWA),SolanoCounty \| 2011 \| Neg_2011_Anasplatyrhynchos \| \| UCD.11.02525.P.H4 \| Pos \| 2 \| 38.35 \| 17 \| 4 \| 6 \| H4N6 \| M \| 12-Aug-11 \| GrizzlyIslandWildlifeArea(GIWA),SolanoCounty \| 2011 \| Pos_2011_Anasplatyrhynchos \| \| UCD.11.02526 \| Neg \| 0 \| NA \| NA \| neg \| neg \| neg \| M \| 12-Aug-11 \| GrizzlyIslandWildlifeArea(GIWA),SolanoCounty \| 2011 \| Neg_2011_Anasplatyrhynchos \| \| UCD.11.02527.V.H5 \| Pos \| 4 \| 28.83 \| 26 \| 5 \| 1 \| H5N1 \| M \| 12-Aug-11 \| GrizzlyIslandWildlifeArea(GIWA),SolanoCounty \| 2011 \| Pos_2011_Anasplatyrhynchos \| \| UCD.11.02528 \| Neg \| 0 \| NA \| NA \| neg \| neg \| neg \| F \| 12-Aug-11 \| GrizzlyIslandWildlifeArea(GIWA),SolanoCounty \| 2011 \| Neg_2011_Anasplatyrhynchos \| \| UCD.11.02529 \| Neg \| 0 \| NA \| NA \| neg \| neg \| neg \| M \| 12-Aug-11 \| GrizzlyIslandWildlifeArea(GIWA),SolanoCounty \| 2011 \| Neg_2011_Anasplatyrhynchos \| \| UCD.11.02531.P.H5 \| Pos \| 3 \| 30.5 \| 26 \| 5 \| 1 \| H5N1 \| M \| 12-Aug-11 \| GrizzlyIslandWildlifeArea(GIWA),SolanoCounty \| 2011 \| Pos_2011_Anasplatyrhynchos \| \| UCD.11.02532.H4 \| Pos \| 1 \| 41.6 \| 18 \| 4 \| 6 \| H4N6 \| M \| 12-Aug-11 \| GrizzlyIslandWildlifeArea(GIWA),SolanoCounty \| 2011 \| Pos_2011_Anasplatyrhynchos \| \| UCD.11.02533.V.H4 \| Pos \| 3 \| 31.47 \| 17 \| 4 \| 6 \| H4N6 \| M \| 12-Aug-11 \| GrizzlyIslandWildlifeArea(GIWA),SolanoCounty \| 2011 \| Pos_2011_Anasplatyrhynchos \| \| UCD.11.02535.P.H5 \| Pos \| 3 \| 31.96 \| 26 \| 5 \| 1 \| H5N1 \| M \| 12-Aug-11 \| GrizzlyIslandWildlifeArea(GIWA),SolanoCounty \| 2011 \| Pos_2011_Anasplatyrhynchos \| \| UCD.11.02536.H5 \| Pos \| 3 \| 30.17 \| 26 \| 5 \| 1 \| H5N1 \| M \| 12-Aug-11 \| GrizzlyIslandWildlifeArea(GIWA),SolanoCounty \| 2011 \| Pos_2011_Anasplatyrhynchos \| \| UCD.11.02538 \| Neg \| 0 \| NA \| NA \| neg \| neg \| neg \| F \| 12-Aug-11 \| GrizzlyIslandWildlifeArea(GIWA),SolanoCounty \| 2011 \| Neg_2011_Anasplatyrhynchos \| \| UCD.11.02541 \| Neg \| 0 \| NA \| NA \| neg \| neg \| neg \| M \| 12-Aug-11 \| GrizzlyIslandWildlifeArea(GIWA),SolanoCounty \| 2011 \| Neg_2011_Anasplatyrhynchos \| \| UCD.11.02542.V.H5 \| Pos \| NA \| NA \| 26 \| 5 \| 1 \| H5N1 \| M \| 12-Aug-11 \| GrizzlyIslandWildlifeArea(GIWA),SolanoCounty \| 2011 \| Pos_2011_Anasplatyrhynchos \| \| UCD.11.02546 \| Neg \| 0 \| NA \| NA \| neg \| neg \| neg \| F \| 12-Aug-11 \| GrizzlyIslandWildlifeArea(GIWA),SolanoCounty \| 2011 \| Neg_2011_Anasplatyrhynchos \| \| UCD.11.02547.V.H3 \| Pos \| 2 \| 36.74 \| 6 \| 3 \| 8 \| H3N8 \| M \| 12-Aug-11 \| GrizzlyIslandWildlifeArea(GIWA),SolanoCounty \| 2011 \| Pos_2011_Anasplatyrhynchos \| \| UCD.11.02549.V.H3 \| Pos \| 1 \| 40.55 \| 6 \| 3 \| 8 \| H3N8 \| F \| 12-Aug-11 \| GrizzlyIslandWildlifeArea(GIWA),SolanoCounty \| 2011 \| Pos_2011_Anasplatyrhynchos \| \| UCD.11.02552.V.H4 \| Pos \| 2 \| 37.23 \| 22 \| 4 \| 8 \| H4N8 \| M \| 12-Aug-11 \| GrizzlyIslandWildlifeArea(GIWA),SolanoCounty \| 2011 \| Pos_2011_Anasplatyrhynchos \| \| UCD.11.02563.P.H4 \| Pos \| 3 \| 30.82 \| 18 \| 4 \| 6 \| H4N6 \| F \| 12-Aug-11 \| GrizzlyIslandWildlifeArea(GIWA),SolanoCounty \| 2011 \| Pos_2011_Anasplatyrhynchos \| \| UCD.11.02566.P.H4 \| Pos \| 2 \| 35.3 \| 23 \| 4 \| 8 \| H4N8 \| F \| 12-Aug-11 \| GrizzlyIslandWildlifeArea(GIWA),SolanoCounty \| 2011 \| Pos_2011_Anasplatyrhynchos \| \| UCD.11.02584.P.H4 \| Pos \| 1 \| 42.61 \| 24 \| 4 \| 9 \| H4N9 \| M \| 12-Aug-11 \| GrizzlyIslandWildlifeArea(GIWA),SolanoCounty \| 2011 \| Pos_2011_Anasplatyrhynchos \| \| UCD.11.02589.P.H4 \| Pos \| 2 \| 35.4 \| 11 \| 4 \| 1 \| H4N1 \| M \| 12-Aug-11 \| GrizzlyIslandWildlifeArea(GIWA),SolanoCounty \| 2011 \| Pos_2011_Anasplatyrhynchos \| \| UCD.11.02590.V.H4 \| Pos \| 2 \| 35.06 \| 19 \| 4 \| 6 \| H4N6 \| M \| 12-Aug-11 \| GrizzlyIslandWildlifeArea(GIWA),SolanoCounty \| 2011 \| Pos_2011_Anasplatyrhynchos \| \| UCD.11.02594.P.H4 \| Pos \| 3 \| 34.83 \| 18 \| 4 \| 6 \| H4N6 \| F \| 12-Aug-11 \| GrizzlyIslandWildlifeArea(GIWA),SolanoCounty \| 2011 \| Pos_2011_Anasplatyrhynchos \| \| UCD.12.02721.P \| Neg \| 0 \| NA \| NA \| neg \| neg \| neg \| F \| 9-Jul-12 \| GrizzlyIslandWildlifeArea(GIWA),SolanoCounty \| 2012 \| Neg_2012_Anasplatyrhynchos \| \| UCD.12.02722.P \| Neg \| 0 \| NA \| NA \| neg \| neg \| neg \| M \| 9-Jul-12 \| GrizzlyIslandWildlifeArea(GIWA),SolanoCounty \| 2012 \| Neg_2012_Anasplatyrhynchos \| \| UCD.12.02723.P \| Neg \| 0 \| NA \| NA \| neg \| neg \| neg \| U \| 9-Jul-12 \| GrizzlyIslandWildlifeArea(GIWA),SolanoCounty \| 2012 \| Neg_2012_Anasplatyrhynchos \| \| UCD.12.02724.P \| Neg \| 0 \| NA \| NA \| neg \| neg \| neg \| M \| 9-Jul-12 \| GrizzlyIslandWildlifeArea(GIWA),SolanoCounty \| 2012 \| Neg_2012_Anasplatyrhynchos \| \| UCD.12.02725.P \| Neg \| 0 \| NA \| NA \| neg \| neg \| neg \| M \| 9-Jul-12 \| GrizzlyIslandWildlifeArea(GIWA),SolanoCounty \| 2012 \| Neg_2012_Anasplatyrhynchos \| \| UCD.12.02726.P \| Neg \| 0 \| NA \| NA \| neg \| neg \| neg \| F \| 9-Jul-12 \| GrizzlyIslandWildlifeArea(GIWA),SolanoCounty \| 2012 \| Neg_2012_Anasplatyrhynchos \| \| UCD.12.02727.P \| Neg \| 0 \| NA \| NA \| neg \| neg \| neg \| M \| 9-Jul-12 \| GrizzlyIslandWildlifeArea(GIWA),SolanoCounty \| 2012 \| Neg_2012_Anasplatyrhynchos \| \| UCD.12.02729.P \| Neg \| 0 \| NA \| NA \| neg \| neg \| neg \| F \| 9-Jul-12 \| GrizzlyIslandWildlifeArea(GIWA),SolanoCounty \| 2012 \| Neg_2012_Anasplatyrhynchos \| \| UCD.12.02730.P \| Neg \| 0 \| NA \| NA \| neg \| neg \| neg \| F \| 9-Jul-12 \| GrizzlyIslandWildlifeArea(GIWA),SolanoCounty \| 2012 \| Neg_2012_Anasplatyrhynchos \| \| UCD.12.02743.VB.H3 \| Pos \| 2 \| 38.78 \| 7 \| 3 \| 8 \| H3N8 \| F \| 9-Jul-12 \| GrizzlyIslandWildlifeArea(GIWA),SolanoCounty \| 2012 \| Pos_2012_Anasplatyrhynchos \| \| UCD.12.02744.VB.H3 \| Pos \| 2 \| 35.55 \| 7 \| 3 \| 8 \| H3N8 \| F \| 11-Jul-12 \| GrizzlyIslandWildlifeArea(GIWA),SolanoCounty \| 2012 \| Pos_2012_Anasplatyrhynchos \| \| UCD.12.02762.VA.H3 \| Pos \| 2 \| 36.66 \| 7 \| 3 \| 8 \| H3N8 \| F \| 11-Jul-12 \| GrizzlyIslandWildlifeArea(GIWA),SolanoCounty \| 2012 \| Pos_2012_Anasplatyrhynchos \| \| UCD.12.02783.VA.H3 \| Pos \| 2 \| 39.92 \| 9 \| 3 \| 8 \| H3N8 \| M \| 13-Jul-12 \| GrizzlyIslandWildlifeArea(GIWA),SolanoCounty \| 2012 \| Pos_2012_Anasplatyrhynchos \| \| UCD.12.02829.VA.H2 \| Pos \| 3 \| 34.16 \| 1 \| 2 \| 3 \| H2N3 \| M \| 16-Jul-12 \| GrizzlyIslandWildlifeArea(GIWA),SolanoCounty \| 2012 \| Pos_2012_Anasplatyrhynchos \| \| UCD.12.02833.VA.H2 \| Pos \| 3 \| 33.41 \| 1 \| 2 \| 3 \| H2N3 \| M \| 16-Jul-12 \| GrizzlyIslandWildlifeArea(GIWA),SolanoCounty \| 2012 \| Pos_2012_Anasplatyrhynchos \| \| UCD.12.02838.VB.H2 \| Pos \| 2 \| 35.26 \| 1 \| 2 \| 3 \| H2N3 \| M \| 16-Jul-12 \| GrizzlyIslandWildlifeArea(GIWA),SolanoCounty \| 2012 \| Pos_2012_Anasplatyrhynchos \| \| UCD.12.02839.VB.H2 \| Pos \| 3 \| 30.7 \| 2 \| 2 \| 3 \| H2N3 \| F \| 16-Jul-12 \| GrizzlyIslandWildlifeArea(GIWA),SolanoCounty \| 2012 \| Pos_2012_Anasplatyrhynchos \| \| UCD.12.02840.VB.H2 \| Pos \| 3 \| 33.83 \| 2 \| 2 \| 3 \| H2N3 \| M \| 16-Jul-12 \| GrizzlyIslandWildlifeArea(GIWA),SolanoCounty \| 2012 \| Pos_2012_Anasplatyrhynchos \| \| UCD.12.02841.VA.H2 \| Pos \| 1 \| 40.36 \| 2 \| 2 \| 3 \| H2N3 \| M \| 16-Jul-12 \| GrizzlyIslandWildlifeArea(GIWA),SolanoCounty \| 2012 \| Pos_2012_Anasplatyrhynchos \| \| UCD.12.02858.VB.H2 \| Pos \| 3 \| 34.48 \| 4 \| 2 \| 7 \| H2N7 \| M \| 18-Jul-12 \| GrizzlyIslandWildlifeArea(GIWA),SolanoCounty \| 2012 \| Pos_2012_Anasplatyrhynchos \| \| UCD.12.02867.VB.H2 \| Pos \| 3 \| 31.38 \| 2 \| 2 \| 3 \| H2N3 \| M \| 18-Jul-12 \| GrizzlyIslandWildlifeArea(GIWA),SolanoCounty \| 2012 \| Pos_2012_Anasplatyrhynchos \| \| UCD.12.02885.VB.H2 \| Pos \| 4 \| 29.43 \| 3 \| 2 \| 3 \| H2N3 \| M \| 20-Jul-12 \| GrizzlyIslandWildlifeArea(GIWA),SolanoCounty \| 2012 \| Pos_2012_Anasplatyrhynchos \| \| UCD.12.02904.VB.H3 \| Pos \| 2 \| 39.01 \| 9 \| 3 \| 8 \| H3N8 \| M \| 20-Jul-12 \| GrizzlyIslandWildlifeArea(GIWA),SolanoCounty \| 2012 \| Pos_2012_Anasplatyrhynchos \| \| UCD.12.02924.VB.H3 \| Pos \| 2 \| 39.45 \| 8 \| 3 \| 8 \| H3N8 \| F \| 23-Jul-12 \| GrizzlyIslandWildlifeArea(GIWA),SolanoCounty \| 2012 \| Pos_2012_Anasplatyrhynchos \| \| UCD.12.02945.VB.H3 \| Pos \| 2 \| 36.68 \| 10 \| 3 \| 8 \| H3N8 \| F \| 23-Jul-12 \| GrizzlyIslandWildlifeArea(GIWA),SolanoCounty \| 2012 \| Pos_2012_Anasplatyrhynchos \| \| UCD.13.01343 \| Neg \| 0 \| NA \| NA \| neg \| neg \| neg \| F \| 18-Jul-13 \| GrizzlyIslandWildlifeArea(GIWA),SolanoCounty \| 2013 \| Neg_2013_Anasplatyrhynchos \| \| UCD.13.01344 \| Neg \| 0 \| NA \| NA \| neg \| neg \| neg \| M \| 18-Jul-13 \| GrizzlyIslandWildlifeArea(GIWA),SolanoCounty \| 2013 \| Neg_2013_Anasplatyrhynchos \| \| UCD.13.01345 \| Neg \| 0 \| NA \| NA \| neg \| neg \| neg \| F \| 18-Jul-13 \| GrizzlyIslandWildlifeArea(GIWA),SolanoCounty \| 2013 \| Neg_2013_Anasplatyrhynchos \| \| UCD.13.01346 \| Neg \| 0 \| NA \| NA \| neg \| neg \| neg \| F \| 18-Jul-13 \| GrizzlyIslandWildlifeArea(GIWA),SolanoCounty \| 2013 \| Neg_2013_Anasplatyrhynchos \| \| UCD.13.01347 \| Neg \| 0 \| NA \| NA \| neg \| neg \| neg \| M \| 18-Jul-13 \| GrizzlyIslandWildlifeArea(GIWA),SolanoCounty \| 2013 \| Neg_2013_Anasplatyrhynchos \| \| UCD.13.01349.VB.H4 \| Pos \| 2 \| 36.77 \| 20 \| 4 \| 6 \| H4N6 \| F \| 22-Jul-13 \| GrizzlyIslandWildlifeArea(GIWA),SolanoCounty \| 2013 \| Pos_2013_Anasplatyrhynchos \| \| UCD.13.01351 \| Neg \| 0 \| NA \| NA \| neg \| neg \| neg \| F \| 22-Jul-13 \| GrizzlyIslandWildlifeArea(GIWA),SolanoCounty \| 2013 \| Neg_2013_Anasplatyrhynchos \| \| UCD.13.01352 \| Neg \| 0 \| NA \| NA \| neg \| neg \| neg \| F \| 22-Jul-13 \| GrizzlyIslandWildlifeArea(GIWA),SolanoCounty \| 2013 \| Neg_2013_Anasplatyrhynchos \| \| UCD.13.01354 \| Neg \| 0 \| NA \| NA \| neg \| neg \| neg \| F \| 22-Jul-13 \| GrizzlyIslandWildlifeArea(GIWA),SolanoCounty \| 2013 \| Neg_2013_Anasplatyrhynchos \| \| UCD.13.01357.VB.H5 \| Pos \| 3 \| 33.32 \| 27 \| 5 \| 5 \| H5N5 \| F \| 22-Jul-13 \| GrizzlyIslandWildlifeArea(GIWA),SolanoCounty \| 2013 \| Pos_2013_Anasplatyrhynchos \| \| UCD.13.01358.PA.H4 \| Pos \| 1 \| 40.14 \| 21 \| 4 \| 6 \| H4N6 \| M \| 22-Jul-13 \| GrizzlyIslandWildlifeArea(GIWA),SolanoCounty \| 2013 \| Pos_2013_Anasplatyrhynchos \| \| UCD.13.01369.VB.H4 \| Pos \| 3 \| 33.28 \| 21 \| 4 \| 6 \| H4N6 \| F \| 22-Jul-13 \| GrizzlyIslandWildlifeArea(GIWA),SolanoCounty \| 2013 \| Pos_2013_Anasplatyrhynchos \| \| UCD.13.01370.VA.H5 \| Pos \| 4 \| 24.78 \| 28 \| 5 \| 5 \| H5N5 \| F \| 22-Jul-13 \| GrizzlyIslandWildlifeArea(GIWA),SolanoCounty \| 2013 \| Pos_2013_Anasplatyrhynchos \| \| UCD.13.01371.VA.H5 \| Pos \| 2 \| 35.71 \| 28 \| 5 \| 5 \| H5N5 \| F \| 22-Jul-13 \| GrizzlyIslandWildlifeArea(GIWA),SolanoCounty \| 2013 \| Pos_2013_Anasplatyrhynchos \| \| UCD.13.01375.VA.H5 \| Pos \| 3 \| 32.99 \| 28 \| 5 \| 5 \| H5N5 \| F \| 22-Jul-13 \| GrizzlyIslandWildlifeArea(GIWA),SolanoCounty \| 2013 \| Pos_2013_Anasplatyrhynchos \| \| UCD.13.01376.VB.H5 \| Pos \| 3 \| 34.66 \| 28 \| 5 \| 5 \| H5N5 \| F \| 22-Jul-13 \| GrizzlyIslandWildlifeArea(GIWA),SolanoCounty \| 2013 \| Pos_2013_Anasplatyrhynchos \| \| UCD.13.01386.VB.H5 \| Pos \| 0 \| NA \| NA \| 5 \| neg \| neg \| F \| 23-Jul-13 \| GrizzlyIslandWildlifeArea(GIWA),SolanoCounty \| 2013 \| Neg_2013_Anasplatyrhynchos \| \| UCD.13.01392.VB.H5 \| Pos \| 4 \| 25.22 \| 28 \| 5 \| 5 \| H5N5 \| M \| 23-Jul-13 \| GrizzlyIslandWildlifeArea(GIWA),SolanoCounty \| 2013 \| Pos_2013_Anasplatyrhynchos \| \| UCD.13.01395.VB.H5 \| Pos \| 4 \| 28.84 \| 28 \| 5 \| 5 \| H5N5 \| M \| 23-Jul-13 \| GrizzlyIslandWildlifeArea(GIWA),SolanoCounty \| 2013 \| Pos_2013_Anasplatyrhynchos \| \| UCD.13.01396.VB.H4 \| Pos \| 2 \| 38.17 \| 21 \| 4 \| 6 \| H4N6 \| F \| 23-Jul-13 \| GrizzlyIslandWildlifeArea(GIWA),SolanoCounty \| 2013 \| Pos_2013_Anasplatyrhynchos \| \| UCD.13.01397.VB.H5 \| Pos \| 3 \| 31.35 \| 28 \| 5 \| 5 \| H5N5 \| M \| 23-Jul-13 \| GrizzlyIslandWildlifeArea(GIWA),SolanoCounty \| 2013 \| Pos_2013_Anasplatyrhynchos \| \| UCD.13.01398.VB.H4 \| Pos \| 2 \| 36.17 \| 21 \| 4 \| 6 \| H4N6 \| F \| 23-Jul-13 \| GrizzlyIslandWildlifeArea(GIWA),SolanoCounty \| 2013 \| Pos_2013_Anasplatyrhynchos \| \| UCD.13.01399.VB.H5 \| Pos \| 3 \| 32.48 \| 28 \| 5 \| 5 \| H5N5 \| F \| 23-Jul-13 \| GrizzlyIslandWildlifeArea(GIWA),SolanoCounty \| 2013 \| Pos_2013_Anasplatyrhynchos \| \| UCD.13.01483.VB.H4 \| Pos \| 3 \| 30.94 \| 21 \| 4 \| 6 \| H4N6 \| M \| 30-Jul-13 \| GrizzlyIslandWildlifeArea(GIWA),SolanoCounty \| 2013 \| Pos_2013_Anasplatyrhynchos \| \| UCD.13.01486.PA.H4 \| Pos \| 2 \| 39.82 \| 21 \| 4 \| 6 \| H4N6 \| M \| 30-Jul-13 \| GrizzlyIslandWildlifeArea(GIWA),SolanoCounty \| 2013 \| Pos_2013_Anasplatyrhynchos \| |
| --- | --- | --- | --- | --- | --- | --- | --- | --- | --- | --- | --- | --- | --- | --- | --- | --- | --- | --- | --- | --- | --- | --- | --- | --- | --- | --- | --- | --- | --- | --- | --- | --- | --- | --- | --- | --- | --- | --- | --- | --- | --- | --- | --- | --- | --- | --- | --- | --- | --- | --- | --- | --- | --- | --- | --- | --- | --- | --- | --- | --- | --- | --- | --- | --- | --- | --- | --- | --- | --- | --- | --- | --- | --- | --- | --- | --- | --- | --- | --- | --- | --- | --- | --- | --- | --- | --- | --- | --- | --- | --- | --- | --- | --- | --- | --- | --- | --- | --- | --- | --- | --- | --- | --- | --- | --- | --- | --- | --- | --- | --- | --- | --- | --- | --- | --- | --- | --- | --- | --- | --- | --- | --- | --- | --- | --- | --- | --- | --- | --- | --- | --- | --- | --- | --- | --- | --- | --- | --- | --- | --- | --- | --- | --- | --- | --- | --- | --- | --- | --- | --- | --- | --- | --- | --- | --- | --- | --- | --- | --- | --- | --- | --- | --- | --- | --- | --- | --- | --- | --- | --- | --- | --- | --- | --- | --- | --- | --- | --- | --- | --- | --- | --- | --- | --- | --- | --- | --- | --- | --- | --- | --- | --- | --- | --- | --- | --- | --- | --- | --- | --- | --- | --- | --- | --- | --- | --- | --- | --- | --- | --- | --- | --- | --- | --- | --- | --- | --- | --- | --- | --- | --- | --- | --- | --- | --- | --- | --- | --- | --- | --- | --- | --- | --- | --- | --- | --- | --- | --- | --- | --- | --- | --- | --- | --- | --- | --- | --- | --- | --- | --- | --- | --- | --- | --- | --- | --- | --- | --- | --- | --- | --- | --- | --- | --- | --- | --- | --- | --- | --- | --- | --- | --- | --- | --- | --- | --- | --- | --- | --- | --- | --- | --- | --- | --- | --- | --- | --- | --- | --- | --- | --- | --- | --- | --- | --- | --- | --- | --- | --- | --- | --- | --- | --- | --- | --- | --- | --- | --- | --- | --- | --- | --- | --- | --- | --- | --- | --- | --- | --- | --- | --- | --- | --- | --- | --- | --- | --- | --- | --- | --- | --- | --- | --- | --- | --- | --- | --- | --- | --- | --- | --- | --- | --- | --- | --- | --- | --- | --- | --- | --- | --- | --- | --- | --- | --- | --- | --- | --- | --- | --- | --- | --- | --- | --- | --- | --- | --- | --- | --- | --- | --- | --- | --- | --- | --- | --- | --- | --- | --- | --- | --- | --- | --- | --- | --- | --- | --- | --- | --- | --- | --- | --- | --- | --- | --- | --- | --- | --- | --- | --- | --- | --- | --- | --- | --- | --- | --- | --- | --- | --- | --- | --- | --- | --- | --- | --- | --- | --- | --- | --- | --- | --- | --- | --- | --- | --- | --- | --- | --- | --- | --- | --- | --- | --- | --- | --- | --- | --- | --- | --- | --- | --- | --- | --- | --- | --- | --- | --- | --- | --- | --- | --- | --- | --- | --- | --- | --- | --- | --- | --- | --- | --- | --- | --- | --- | --- | --- | --- | --- | --- | --- | --- | --- | --- | --- | --- | --- | --- | --- | --- | --- | --- | --- | --- | --- | --- | --- | --- | --- | --- | --- | --- | --- | --- | --- | --- | --- | --- | --- | --- | --- | --- | --- | --- | --- | --- | --- | --- | --- | --- | --- | --- | --- | --- | --- | --- | --- | --- | --- | --- | --- | --- | --- | --- | --- | --- | --- | --- | --- | --- | --- | --- | --- | --- | --- | --- | --- | --- | --- | --- | --- | --- | --- | --- | --- | --- | --- | --- | --- | --- | --- | --- | --- | --- | --- | --- | --- | --- | --- | --- | --- | --- | --- | --- | --- | --- | --- | --- | --- | --- | --- | --- | --- | --- | --- | --- | --- | --- | --- | --- | --- | --- | --- | --- | --- | --- | --- | --- | --- | --- | --- | --- | --- | --- | --- | --- | --- | --- | --- | --- | --- | --- | --- | --- | --- | --- | --- | --- | --- | --- | --- | --- | --- | --- | --- | --- | --- | --- | --- | --- | --- | --- | --- | --- | --- | --- | --- | --- | --- | --- | --- | --- | --- | --- | --- | --- | --- | --- | --- | --- | --- | --- | --- | --- | --- | --- | --- | --- | --- | --- | --- | --- | --- | --- | --- | --- | --- | --- | --- | --- | --- | --- | --- | --- | --- | --- | --- | --- | --- | --- | --- | --- | --- | --- | --- | --- | --- | --- | --- | --- | --- | --- | --- | --- | --- | --- | --- | --- | --- | --- | --- | --- | --- | --- | --- | --- | --- | --- | --- | --- | --- | --- | --- | --- | --- | --- | --- | --- | --- | --- | --- | --- | --- | --- | --- | --- | --- | --- | --- | --- | --- | --- | --- | --- | --- | --- | --- | --- | --- | --- | --- | --- | --- | --- | --- | --- | --- | --- | --- | --- | --- | --- | --- | --- | --- | --- | --- | --- | --- | --- | --- | --- | --- | --- | --- | --- | --- | --- | --- | --- | --- | --- | --- | --- | --- | --- | --- | --- | --- | --- | --- | --- | --- | --- | --- | --- | --- | --- | --- | --- | --- | --- | --- | --- | --- | --- | --- | --- | --- | --- | --- | --- | --- | --- | --- | --- | --- | --- | --- | --- | --- | --- | --- | --- | --- | --- | --- | --- | --- | --- | --- | --- | --- | --- | --- | --- | --- | --- | --- | --- | --- | --- | --- | --- | --- | --- | --- | --- | --- | --- | --- | --- | --- | --- | --- | --- | --- | --- | --- | --- | --- | --- | --- | --- | --- | --- | --- | --- | --- | --- | --- | --- | --- | --- | --- | --- | --- | --- | --- | --- | --- | --- | --- | --- | --- | --- | --- | --- | --- | --- | --- | --- | --- | --- | --- | --- | --- | --- | --- | --- | --- | --- | --- | --- | --- | --- | --- | --- | --- | --- | --- | --- | --- | --- | --- | --- | --- | --- | --- | --- | --- | --- | --- | --- | --- | --- | --- | --- | --- | --- | --- | --- | --- | --- | --- | --- | --- | --- | --- | --- | --- | --- | --- | --- | --- | --- | --- | --- | --- | --- | --- | --- | --- | --- | --- | --- | --- | --- | --- | --- | --- | --- | --- | --- | --- | --- | --- | --- | --- | --- | --- | --- | --- | --- | --- | --- | --- | --- | --- | --- | --- | --- | --- | --- | --- | --- | --- | --- | --- | --- | --- | --- | --- | --- | --- | --- | --- | --- | --- | --- | --- | --- | --- | --- | --- | --- | --- | --- | --- | --- | --- | --- | --- | --- | --- | --- | --- | --- | --- | --- | --- | --- | --- | --- | --- | --- | --- | --- | --- | --- | --- | --- | --- | --- | --- | --- | --- | --- | --- | --- | --- | --- | --- | --- | --- | --- | --- | --- | --- | --- | --- | --- | --- | --- | --- | --- | --- | --- | --- | --- | --- | --- | --- | --- | --- | --- | --- | --- | --- | --- | --- | --- | --- | --- | --- | --- | --- | --- | --- | --- | --- | --- | --- | --- | --- | --- | --- | --- | --- | --- | --- | --- | --- | --- | --- | --- | --- | --- | --- | --- | --- | --- | --- | --- | --- | --- | --- | --- | --- | --- | --- | --- | --- | --- | --- | --- | --- | --- | --- | --- | --- | --- | --- | --- | --- | --- | --- | --- | --- | --- | --- | --- | --- | --- | --- | --- | --- | --- | --- | --- | --- | --- | --- | --- | --- | --- | --- | --- | --- | --- | --- | --- | --- | --- | --- | --- | --- | --- | --- | --- | --- | --- | --- | --- | --- | --- | --- | --- | --- | --- | --- | --- | --- | --- | --- | --- | --- | --- | --- | --- | --- | --- | --- | --- | --- | --- | --- | --- | --- | --- | --- | --- | --- | --- | --- | --- | --- | --- | --- | --- | --- | --- | --- | --- | --- | --- | --- | --- | --- | --- | --- | --- | --- | --- | --- | --- | --- | --- | --- | --- | --- | --- | --- | --- | --- | --- | --- | --- | --- | --- | --- | --- | --- | --- | --- | --- | --- | --- | --- | --- | --- | --- | --- | --- | --- | --- | --- | --- | --- | --- | --- | --- | --- | --- | --- | --- | --- | --- | --- | --- | --- | --- | --- | --- | --- | --- | --- | --- | --- | --- | --- | --- | --- | --- | --- | --- | --- | --- | --- | --- | --- | --- | --- | --- | --- | --- | --- | --- | --- | --- | --- | --- | --- | --- | --- | --- | --- | --- | --- | --- | --- | --- | --- | --- | --- | --- | --- | --- | --- | --- | --- | --- | --- | --- | --- | --- | --- | --- | --- | --- | --- | --- | --- | --- | --- | --- | --- | --- | --- | --- | --- | --- | --- | --- | --- | --- | --- | --- | --- | --- | --- | --- | --- | --- | --- | --- | --- | --- | --- | --- | --- | --- | --- | --- | --- | --- | --- | --- | --- | --- | --- | --- | --- | --- | --- | --- | --- | --- | --- | --- | --- | --- | --- | --- | --- | --- | --- | --- | --- | --- | --- | --- | --- | --- | --- | --- | --- | --- | --- | --- | --- | --- | --- | --- | --- | --- | --- | --- | --- | --- | --- | --- | --- | --- | --- | --- | --- | --- | --- | --- | --- | --- | --- | --- | --- | --- | --- | --- | --- | --- | --- | --- | --- | --- | --- | --- | --- | --- | --- | --- | --- | --- | --- | --- | --- | --- | --- | --- | --- | --- | --- | --- | --- | --- | --- | --- | --- | --- | --- | --- | --- | --- | --- | --- | --- | --- | --- | --- | --- | --- | --- | --- | --- | --- | --- | --- | --- | --- | --- | --- | --- | --- | --- | --- | --- | --- | --- | --- | --- | --- | --- | --- | --- | --- | --- | --- | --- | --- | --- | --- | --- | --- | --- | --- | --- | --- | --- | --- | --- | --- | --- | --- | --- | --- | --- | --- | --- | --- | --- | --- | --- | --- | --- | --- | --- | --- | --- | --- | --- | --- | --- | --- | --- | --- | --- | --- | --- | --- |
