# Supplementary material for: Community-Level Differences in the Microbiome of Healthy Wild Mallards and Those Infected by Influenza A Viruses
Source: mSystems. 2017 Feb 28;2(1):e00188-16. doi: 10.1128/mSystems.00188-16 (PMC5347185; doi:10.1128/mSystems.00188-16)
Supplement: TEXT S1 [file sys001172081s1.docx]

**Supplementary Information**

Effect of OTU removal on ordination plots

We verified whether the presence of OTUs identified by G-test statistic or DIROM directly affected clustering by infection status in PCoA space. To do this, we removed the 85 OTUs identified by G test significance, or the 47 OTUs identified by DIROM, and observed in both cases that PCoA clusters were abolished if Bray-Curtis distances were computed on abundance values (Figure S1). If the same process was applied to binarized presence/absence versions of the data, PCoA clusters were not strongly affected, indicating that the G-test statistic and DIROM OTUs are differentially enriched between IAV conditions, but not differentially frequent.

We also examined the effect on ordination of removing OTUs identified by network analysis, as well as the overlapping 41 OTUs that were found to be significant across all methods. We first verified that Bray-Curtis distances across the 674 OTUs separated by IAV condition in PCoA space. We removed the seven highly connected OTUs that were uniquely occurring in IAV+, the 60 highly connected OTUs that were occurring in the IAV- network, and the 20 mutual OTUs found to be highly connected in both IAV+ and IAV-. While these OTUs contribute to some of the clustering by IAV condition, we found that removal of the overlapping 41 OTUs that were significant in all methods (G-test, DIROM, network analysis) abolished the PCoA clusters (Figure S1); and, conversely, that IAV condition is discernible using just these 41 overlapping OTUs (Figures S2 and 5)
